# Going beyond work and family: A longitudinal study on the role of leisure in the work–life interplay

**DOI:** 10.1002/job.2098

**Published:** 2016-03-04

**Authors:** Michaela Knecht, Bettina S. Wiese, Alexandra M. Freund

**Affiliations:** ^1^ Department of Psychology University of Zurich Zurich Switzerland; ^2^ University Research Priority Program Dynamics of Healthy Aging University of Zurich Zurich Switzerland; ^3^ Department of Psychology RWTH Aachen University Aachen Germany

**Keywords:** conflict, facilitation, leisure, work, family, subjective well‐being

## Abstract

Going beyond the relation of work and family, the present three‐wave longitudinal study spanning one year assessed different forms of conflict and facilitation between leisure and the life domains work and family and their relation to subjective well‐being. A sample of *N* = 277 employed men and women reported their perceived conflict and facilitation between leisure, work, and family and subjective well‐being. Results suggest that leisure is a source of facilitation for work and family, and, at the same time, a major recipient of conflict from work and family. Moreover, leisure conflict was negatively correlated and leisure facilitation was positively associated with concurrent subjective well‐being. Both conflict and facilitation between all three life domains remained highly stable over the course of one year. Only few and non‐systematic lagged effects were found, indicating that the variance of the stability of the constructs and their relations over time leave little room for longitudinal predictions. Taken together, the study demonstrates that, similar to work–family relations, conflict and facilitation with the leisure domain are also associated with subjective well‐being and remain highly stable over the course of a year in the lives of young and middle‐aged adults. Copyright © 2016 John Wiley & Sons, Ltd.

Most of the literature concerned with “work–life” conflict and facilitation focuses exclusively on the interplay of work and family, although leisure is another key life domain for most people (Newman, Tay, & Diener, 2014). As convincingly shown in a recent meta‐analysis by Kuykendall, Tay, and Ng (2015), leisure activities do not only take up time in our everyday lives but they also contribute to subjective well‐being. However, despite the apparent importance of leisure, this life domain has received little attention in the literature on the relations between different life domains and their associations with subjective well‐being. This is the main purpose of the current study. More specifically, this article extends previous research on the “work–life” relation by including leisure in exploring conflict and facilitation between different life domains and their associations with subjective well‐being. The main research question was whether young and middle‐aged adults experience more conflict or more facilitation between leisure and other life domains (work and family). Moreover, we investigated the associations of these relations with subjective well‐being using a longitudinal design with three measurement points covering the period of one year. The longitudinal design allowed us to investigate the inter‐domain relations and their associations with subjective well‐being over time.

Previous research has repeatedly shown that work‐family conflict is associated with lower subjective well‐being (Amstad, Meier, Fasel, Elfering, & Semmer, 2011), whereas facilitation between work and family is associated with higher subjective well‐being (e.g., Kinnunen, Feldt, Geurts, & Pulkkinen, 2006; Wiese, Seiger, Schmid, & Freund, 2010). Much less is known about the role of leisure for managing the multiple demands of the everyday lives of middle‐aged adults. Does leisure help to “unwind” after a stressful day at work and when faced with family‐related chores and obligations? Or does it add to the burden of having to manage multiple life domains in the face of limited resources such as time and energy? The present study addresses this question. To gain insight into various forms and directions of the relations between work, family, and leisure, we differentiate between different facets and dimensions of inter‐domain conflict and facilitation.

The literature on “work–life” balance is dominated by cross‐sectional studies (Nohe, Meier, Sonntag, & Michel, 2015), and only little is known about the temporal stability of inter‐domain relations and their associations with subjective well‐being over time and their temporal ordering. Questions related to such time‐lagged relations are, for instance, if lower subjective well‐being leads to higher perceptions of conflict, if higher conflict leads to lower subjective well‐being, or both. The current three‐wave longitudinal study allows testing for such lagged effects.

### The role of leisure

Voss (1967, p. 101) defines leisure as “a period of time referred to as discretionary time … when an individual feels no sense of economic, legal, moral, or social compulsion or obligation.” We largely agree with this definition but maintain that commitment to leisure activities (such as singing in a choir or being a member of a sports team) also comes with a certain degree of obligation that might lead to conflict with other life domains. Therefore, following the subjective approach to defining leisure, we take a less restrictive definition of leisure than Voss and leave it up to the person to define what they consider as leisure (Newman et al., 2014).

Although the importance of leisure for recreation and recovery from taxing work‐related demands has been recognized (e.g., Fritz & Sonnentag, 2005), there is a dearth of research that considers the potentially conflicting or facilitating relations of leisure with work and family. The DRAMMA model proposed by Newman et al. (2014) proposes five psychological mechanisms of how leisure might influence subjective well‐being, namely, through experiencing detachment from work (leading to recovery), autonomy, mastery, meaning, and affiliation. Whereas four of these five mechanisms describe experiences associated with engaging in specific leisure activities (e.g., feelings of mastery when playing golf), detachment‐recovery inherently involves the relation to other life domains. Detachment‐recovery denotes the process that engaging in leisure activities helps to detach from work‐related (or other kinds of) stress and preoccupations and, thereby, contributes to recharging overtaxed resources or, in other words, to recovery. Note that detachment is not restricted to take place only to recover from work and family, but it might also be good at times to detach from certain leisure activities (e.g., when feeling overwhelmed from and preoccupied with planning a vacation in an exotic country). For such taxing and exhausting leisure activities, work or family might actually help to detach and recover from them.

According to the DRAMMA model, leisure seems to be primarily a life domain that facilitates functioning in the life domains of work and family. However, engaging in leisure activities also takes time, often requires energy, and sometimes also money, which might conflict with pursuing goals in the work or family domain. This might also add to the many demands that juggling work and family already pose in the everyday lives of young and middle‐aged adults. In short, then, we maintain that including leisure in the study of “work–life” balance provides a more comprehensive picture of the inter‐domain relations than considering only work and family.

### Conflict between life domains

The ample literature on the interplay of work and family has convincingly shown that these two life domains place high demands on young and middle‐aged adults who are in a life phase that requires the investment of time and energy in furthering one's career as well as in spending time with one's partner and children (for an overview, see Eby, Caspar, Lockwood, Bordeaux, & Brinley, 2005; Wiese & Freund, 2000). Engagement in leisure activities draws on some of the same resources as work and family do (e.g., time and energy). Not surprisingly, young and middle adulthood has been called the “rush hour” of life (Freund, Nikitin, & Ritter, 2009). Conflict between the life domains can be internally or externally generated (Carlson & Frone, 2003). Internally generated conflict describes a “psychological preoccupation with one domain of life (e.g., work) while within the role boundaries of another domain of life (e.g., family), such as when one cannot stop thinking or ruminating about work when he or she is at home” (Carlson & Frone, 2003, p. 518). External conflict occurs when demands of one life domain prevent participation in another life domain. In other words, externally generated conflict emerges through behavioral involvement (e.g., by investing time), whereas internally generated conflict emerges through psychological involvement (e.g., by being cognitively preoccupied with one life domain). These two forms of conflict might exist simultaneously such as when one spends a lot of time preparing for a marathon instead of spending the time on work‐related projects or spending time with one's family (as a form of externally generated conflict) while also thinking about marathon preparation when at work or during family meals (as a form of internally generated conflict).

Conflict between life domains may originate in either domain (i.e., they are bidirectional; Greenhaus & Beutell, 1985). For example, leisure activities might be organized in a way that they do not conflict with work hours, but preoccupation with work might interfere with leisure activities. The different directions of conflict are not equally frequently reported: more work often interferes with private life than vice versa (e.g., Frone, Russell, & Cooper, 1992).

### Facilitation between life domains

Within the last 15 years, the positive aspects of the interplay between work and family have come more and more into the focus of research (e.g., Grzywacz & Marks, 2000). Life domains may profit from each other in different ways. As is true for conflict, facilitation between life domains might originate in either life domain. Wiese et al. (2010) delineated three facets of facilitation, namely, (1) transfer of positive mood, (2) transfer of competencies from one domain to another, and (3) compensation. *Compensation* describes a buffering effect of engagement in another life domain after a negative experience in a different life domain. For instance, family might take one's mind off a work‐related setback and thereby help to distance oneself emotionally from the negative event (Wiese et al., 2010). These different forms of facilitation are not exhaustive but they cover significant aspects in the interplay between life domains (for an overview of more mechanisms linking work and family, see Edwards & Rothbard, 2000). As is true for the different forms of conflict, these different forms of facilitation can also occur simultaneously.

### The interplay of leisure, work, and family

There is evidence that leisure activities are not independent from the other life domains. For instance, leisure activities are influenced by the employment status (Le Feuvre, 1994). Furthermore, leisure activities impact on work performance (Fritz & Sonnentag, 2005). In situations of inter‐domain conflict, people have an increased need for recovery (Demerouti, Taris, & Bakker, 2007) but reduced opportunities for recovery (Taris et al., 2006). This might be the case because work‐related and family‐related demands are perceived as obligations, whereas—by definition—leisure activities are mostly optional. Thus, if the demands of family and work are very high, leisure activities might be perceived as the life domain from which it is easiest to disengage. Consequently, leisure might show less conflict with the other life domains (as people might give it up more easily when conflict occurs) and more facilitation (as it helps to recover from stress or negative experiences in the other life domains).

Taken together, the present study investigates the following three hypotheses:

*Hypothesis 1*: Leisure is perceived less as a source of conflict (internal and external) than work and family.
*Hypothesis 2*: Leisure receives more conflict (internal and external) than work and family.
*Hypothesis 3*: Leisure is perceived more as a source of facilitation (all three forms) than work and family.

### Associations between inter‐domain relations and subjective well‐being

#### Conflict and subjective well‐being

Studies investigating the effect of conflict between work and family or between work and “the rest of life” (i.e., non‐work) on subjective well‐being have consistently shown a negative association (Amstad et al., 2011). We expect a similar negative association with subjective well‐being also for leisure conflict resulting in the following hypothesis:

*Hypothesis 4*: Conflict between leisure and work or family is negatively associated with subjective well‐being.

Does conflict cause a decrease in subjective well‐being or does lower subjective well‐being lead people to report more conflict? Cross‐sectional studies cannot address the issue of causality, and the results of the few longitudinal studies are mixed, providing some evidence for both causal relations (for an overview, see Steinmetz, Frese, & Schmidt, 2008). Note, however, that two studies did not find evidence for any longitudinal effects: Neither Rantanen, Kinnunen, Feldt, and Pulkkinen (2008) nor O'Driscoll, Brough, and Kalliath (2004) found any cross‐lagged effects between work‐family conflict and psychological well‐being. In a recent meta‐analysis, Nohe et al. (2015) showed a reciprocal effect between work‐family conflict and strain. Given this mixed empirical evidence, there is clearly a need for more studies testing cross‐lagged relations in both directions between inter‐domain conflict and subjective well‐being.

Taken together, the inconsistent results do not allow to formulate a directed hypothesis but instead lead to the following research question:

RQ1: Are there cross‐lagged effects of conflict on subjective well‐being and vice versa that could reveal information about possible causal relationships?

#### Facilitation and subjective well‐being

Mirror imaging the concurrent relations of conflict and subjective well‐being, facilitation between life domains has been shown to be positively associated with well‐being (e.g., Freund, Knecht, & Wiese, 2014; Greenhaus & Allen, 2011; Wiese et al., 2010). We expect a similar positive association with subjective well‐being for facilitation with the leisure domain:

*Hypothesis 5*: Facilitation between leisure with work or family is positively associated with subjective well‐being.

McNall, Nicklin, and Masuda (2009) found in a meta‐analytic review support for an association between work‐family facilitation and physical and mental health. Longitudinal research addressing the causal direction of this association is scarce. The few existing studies show diverging results. Hammer, Cullen, Neal, Sinclair, and Shafiro (2005) did not find any longitudinal effects between work‐family facilitation and depression, whereas Langballe, Innstrand, Aasland, and Falkum (2011) found some longitudinal effects of work‐home facilitation on well‐being, and Innstrand, Langballe, Espens, Falkum, and Aasland (2008) reported evidence for reciprocal effects between work–family facilitation and depression. These mixed results lead to the second research question of the current study:

RQ2: Are there cross‐lagged effects of facilitation on subjective well‐being and vice versa that could reveal information about possible causal relationships?

## Method

In this three‐wave longitudinal study, we investigated different facets and dimensions of perceived conflict and facilitation between work, family, and leisure and their associations with subjective well‐being in young and middle‐aged adults over a period of one year. The time between the measurement occasions was six months.

### Data collection

Data were collected using an online questionnaire. Participants were recruited in the German‐speaking part of Switzerland mostly from the region of Zurich by contacting companies, via newspaper article, through a newsletter for small‐sized and medium‐sized companies, and through flyers in commercial buildings. Criteria for participation were that participants had to (1) be between 30 and 55 years of age, (2) work more than 20 h per week, and (3) live with their partner and/or have children. With these criteria, we wanted to make sure that participants had demands both in the work and in the family domain. To ensure that participants were also engaged in the leisure domain, we asked them to list their two most important leisure‐related goals in addition to two work‐related and family‐related goals. Examples of leisure goals are “to do more sports,” “to meet friends regularly,” or “to learn a foreign language.” Only participants who were able to list two leisure‐related goals in addition to two work‐related and family‐related goals were included in the study (a total of six goals was mandatory). Participants received a reimbursement of 60 Swiss francs (which corresponds to about 60 US dollars) for participation in all three waves. They could choose between a bank transfer, an Amazon voucher, or a donation for “Doctors Without Borders.”

### Sample

The initial sample consisted of a group of *N* = 277 employed young and middle‐aged adults living with their family or partner. Their age ranged from 30 to 55 years (*M* = 41.76, *SD* = 7.19); 57% of the sample were women, 30.8% of the sample did not have children, 19.6% had one child, 33.3% had two children, and 16.3% had three or more children. Regarding education, 34.3% held a university degree and 15.9% went to an applied college. All of the participants were employed, but only 61.0% of them worked fulltime and 39% worked part‐time. More women (57.3%) than men (15.8%) worked part‐time. About half of the sample lived in a household with their partner and child(ren) (51.3%), about one third lived together with their partner (35.7%), 9% as single parents, very few in a multigenerational household (1.1%), or in a living community with others (1.4%).

The sample attrition over time was fairly low with *n* = 253 (91%) at T2 and *n* = 248 (90%) at T3 staying in the study. *N* = 237 (86%) provided data at all three measurement occasions. Selectivity analyses showed that participants who dropped out did not significantly differ from continued participants concerning socio‐demographic variables, conflict, or facilitation (all *t*(271) < 1.50, all *p* > .13). Dropouts reported significant lower general life satisfaction than participants who participated in all three waves, *t*(273) = 2.96, *p* = .003, *d* = 0.48. No significant differences were found for the other well‐being measures (mood and psychosomatic complaints; see description of the measures in the succeeding sections).

### Measures

We used identical measures at all three measurement points. If not noted otherwise, the response scales ranged from 0 (*not at all* or *never*, respectively) to 5 (*very much* or *always*, respectively).

#### Inter‐domain relations

To measure relations between leisure, family, and work, we assessed two forms of conflict and three forms of facilitation.

##### Conflict

Conflict between the life domains was assessed using an adapted version of the scale by Carlson and Frone (2003; German translation by Seiger & Wiese, 2009, items received from the authors), which differentiates between internal and external conflict between work and family. The scale was extended to the leisure domain and shortened to 36 items. A sample item for external family‐to‐leisure conflict reads: “How often does your family life/partnership keep you from spending the amount of time for leisure you would like?” The items of the entire scale are listed in the Supporting Information Appendix 1. Confirmatory factor analyses showed that a 12‐factor solution with correlated errors for the items with the same wording for different life domains best fit the data (Table 1). This indicates that the different directions and different forms of conflict (internal versus external) should be analyzed separately. The same 12‐factor solution was also the best fit to the data of T2 and T3 (not shown in the paper). The 12‐factor solution is consistent with the original scale by Carlson and Frone (2003).

**Table 1:** Confirmatory factor analyses of the life domain conflict scale.

Model	Chi^2^	*df*	CFI	RMSEA	Δ Chi^2^ (Δ *df*)
12 factors^a^ (correlated errors)	704.36	438	0.960	0.047	
12 factors^a^	1065.30	528	0.920	0.061	360.94 (90) ***
6 factors b	3818.36	579	0.518	0.142	3114.00 (141)***
3 factors^c^	4206.15	591	0.463	0.149	3501.79 (153)***
1 factor^d^	5509.45	594	0.269	0.173	4805.09 (156)***

*Note*:

a One factor of each form of conflict (internal/external) for each pair of life domains.

b One factor for each pair of life domain conflict.

c One factor for each life domain as source.

d One factor for all conflict items.

* *p* ≤ .05.

** *p* < .01.

*** *p* ≤ .001.

##### Facilitation

Facilitation between life domains was assessed with the scale from Wiese et al. (2010) with the subscales of transfer of positive mood, transfer of competencies, and compensation between life domains. We extended this scale to the leisure domain and shortened it to 58 items. A sample item for leisure‐to‐work transfer of positive mood reads: “If I feel good during leisure time, I am in a good mood at work, too.” The items of the entire scale are listed in the Supporting Information Appendix 2. Confirmatory factor analyses showed that an 18‐factor solution with correlated errors for the items with the same wording for different life domains best fit the data (Table 2). The same 18‐factor solution also fit best the data of T2 and T3 (not shown in the paper). This is in line with the findings by Wiese et al. (2010) and indicates that the different aspects and the different directions of facilitation between the life domains should be distinguished.

**Table 2:** Confirmatory factor analyses of the life domain facilitation scale.

Model	Chi^2^	*df*	CFI	RMSEA	Δ Chi^2^(Δ *df*)
18 factors^a^ (correlated errors)	1572.29	1089	0.960	0.040	
18 factors^a^	2523.33	1224	0.891	0.062	951.04 (135) ***
6 factors^b^	8088.33	1362	0.438	0.134	6516.04 (273)***
3 factors c	8459.82	1374	0.408	0.136	6887.53 (285)***
1 factor d	10029.59	1377	0.277	0.151	8457.30 (288)***

*Note*:

a One factor of each form of facilitation (transfer of mood/transfer of competencies/compensation) for each pair of life domains.

b One factor for each pair of life domain facilitations.

c One factor for each life domain as source.

d One factor for all facilitation items.

* *p* ≤ .05.

** *p* < .01.

*** *p* ≤ .001.

Means, standard deviations, intercorrelations, and Cronbach's alphas for the subscales of conflict and facilitation are provided for T1 in the Supporting Information Appendix 3, for T2 in the Supporting Information Appendix 4, and for T3 in the Supporting Information Appendix 5.

#### Subjective well‐being

Three indicators of subjective well‐being were included in the present study. One of these factors taps into the evaluative facet of subjective well‐being (general life satisfaction), one into the affective facet (mood), and one into the health‐related facet of well‐being (self‐reported psychosomatic symptoms).

##### General life satisfaction

Life satisfaction was assessed with the German version (Schumacher, Klaiberg, & Brähler, 2003) of the 5‐item scale by Diener, Emmons, Larsen, and Griffin (1985). At T1, Cronbach's alpha = .86, *M* = 3.38, *SD* = 0.90; at T2, Cronbach's alpha = .87, *M* = 3.44, *SD* = 0.90; at T3, Cronbach's alpha = .90, *M* = 3.44, *SD* = 0.90.

##### Mood

Mood was assessed with the 12‐item version of the Multidimensional Mood Questionnaire (MDMQ; Steyer, Schwenkmezger, Notz, & Eid, 1994). Items reflecting negative emotions were reversed in order to be able to aggregate across all items of the scale. Internal consistencies were good (at T1, *α* = .91, *M* = 3.02, *SD* = 0.82; at T2, *α* = .92, *M* = 3.01, *SD* = 0.84; at T3, *α* = .93, *M* = 3.03, *SD* = 0.85).

##### Self‐reported psychosomatic symptoms

Psychosomatic symptoms were assessed by a slightly modified version of the symptom checklist from the Symptom Checklist‐90‐Revised scale (German version, Franke, 2002). We added one item about sleep disorders, resulting in 13 items assessing psychosomatic complaints such as headaches, back pain, and dizziness. A composite score was built by aggregating the intensity ratings across all complaints (at T1, *α* = .82, *M* = 0.74, *SD* = 0.66; at T2, *α* = .88, *M* = 0.75, *SD* = 0.77; at T3, *α* = .85, *M* = 0.79, *SD* = 0.71).

The three indicators of subjective well‐being were significantly correlated (*r*_general life satisfaction, mood_ = .58, *p* < .01; *r*_general life satisfaction,psychosomatic symptoms (reverse coded)_ = .41, *p* < .01; *r*_mood, psychosomatic complaints (reverse coded)_ = .58, *p* < .01). Thus, for reasons of parsimony, we aggregated them into a higher‐order factor of subjective well‐being in the subsequent analyses. Associations of conflict and facilitation with the different indicators of subjective well‐being were all in the same direction. A table summarizing these relations can be obtained from the first author.

### Data analytic strategy

**Figure 1:**
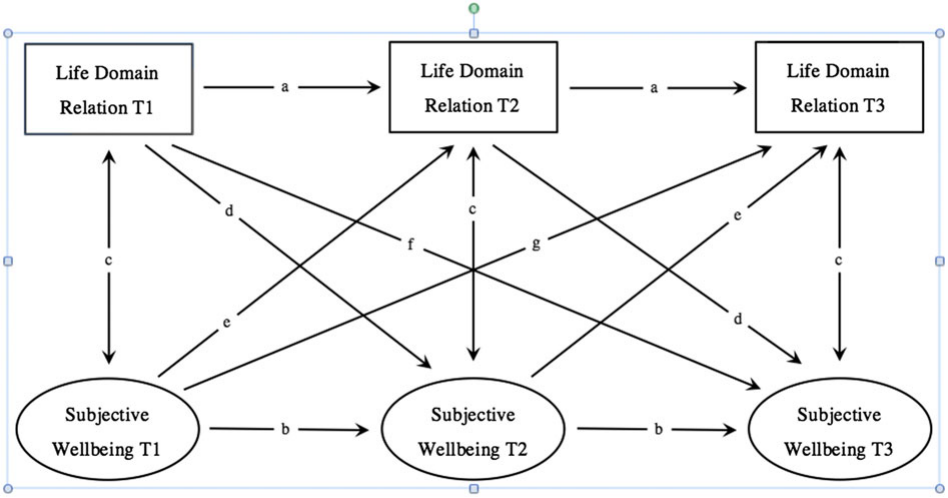
Model of the cross‐lagged panel design depicting the different forms of analysed associations

To investigate the stability of the different facets of inter‐domain relations over time and their cross‐sectional as well as lagged associations with subjective well‐being, a series of structural equation models was conducted with the Lavaan package for R (Rosseel, 2012). Figure 1 depicts the models. There are 12 pairs of dyadic relationships among work, family, and leisure. In the structural equation models, we combined the models for conflict and facilitation between two life domains for each direction. This adds up to six fully cross‐lagged panel designs, two for each pair of life domains, once as a source and once as a recipient of conflict and facilitation. The design of the study allows assessing longitudinal effects of six as well as of 12 months. To facilitate structuring the results, we labeled the different paths of these relations with letters in Figure 1 (stability of life domain relations over six months (a), stability of subjective well‐being over six months (b), synchronous associations between life domain relations and subjective well‐being (c), effects of inter‐domain relations on subjective well‐being with a six months (d) and 12 months (f) time lag, effects of subjective well‐being on inter‐domain relations with a six months (e) and 12 months time lag (g). The letters correspond to the letters of the columns of Table 4). Associations with the same letter were set to be equal. Auto‐covariance for same items was released. For a better model fit, scales were included as observed variables.

## Results

The six structural equation models all showed an acceptable model fit (Table 3). The results of the cross‐lagged panel analyses are listed in Table 4. The letters of the columns correspond to the letters in Figure 1 that show the different associations.

**Table 3:** Model fit of the six three‐wave cross‐lagged panels analyzing the stability of life domain relations and their association with subjective well‐being.

Model	Chi^2^	*df*	CFI	RMSEA
Leisure‐to‐work	146.916	77	0.963	0.057
Leisure‐to‐family	133.072	77	0.969	0.051
Work‐to‐leisure	132.365	77	0.972	0.051
Family‐to‐leisure	146.825	77	0.964	0.057
Work‐to‐family	142.181	77	0.966	0.055
Family‐to‐work	137.235	77	0.969	0.053

Note:

*N* = 277. Robust values reported.

**Table 4:** Overview of stability of life domain conflict and facilitation and their associations with subjective well‐being synchronous and over time.

	(a) Stability over six months	(c) Synchronous associations with subjective well‐being	(d) Six months lagged effect on subjective well‐being	(f) 12 months lagged effects on subjective well‐being	(e) Six months lagged effect of subjective well‐being on life domain relation	(g) 12 months lagged effects of subjective well‐being on the life domain relation
(a) Conflict						
Leisure ➔ work	0.63***/0.66***	−0.03*/−0.03*/−0.03*	−0.06/−0.06	−0.01	−0.07/−0.07	0.05
Leisure ➔ family	0.56***/0.56***	−0.04**/−0.04**/−0.04**	0.01/0.01/	−0.01	−0.07/−0.07	−0.00
Work ➔ leisure	0.64***/0.62***	−0.09***/−0.09***/−0.09***	−0.03/−0.03	0.04*	−0.06/−0.07	−0.07
Family ➔ leisure	0.59***/0.59***	−0.06**/−0.06**/−0.06**	−0.03/−0.02	0.06	−0.15*/−0.17*	0.07
Work ➔ family	0.61***/0.61***	−0.08***/−0.08***/−0.08***	−0.08*/−0.07*	0.15*	−0.09/−0.10	−0.09
Family ➔ work	0.62***/0.70***	−0.07***/−0.07***/−0.07***	−0.03/−0.03	0.01*	−0.04/−0.04	−0.01
(b) Facilitation						
Leisure ➔ work	0.59***/0.60***	0.09***/0.09***/0.09***	−0.01/−0.01	−0.01	0.01/0.01	0.13
Leisure ➔ family	0.55***/0.59***	0.06***/0.06***/0.06***	0.00/0.00	0.02	0.05/0.06	0.02
Work ➔ leisure	0.64***/0.64***	0.04*/0.04*/0.04*	−0.08/−0.07	0.07	0.09/0.10	0.06
Family ➔ leisure	0.58***/0.62***	0.07***/0.07***/0.07***	−0.03/−0.03	0.03	−0.01/−0.01	0.13
Work ➔ family	0.58***/0.59***	0.04**/0.04**/0.04**	−0.03/−0.02	−0.00	0.07/0.08	0.05
Family ➔ work	0.66***/0.64***	0.09***/0.09***/0.09***	−0.03/−0.03	0.03	0.06/0.06	0.05

Note:

*N* = 277. Standardized coefficients.

* *p* ≤ .05.

** *p* < .01.

*** *p* < .001.

### Prevalence of the different forms of conflict

**Figure 2:**
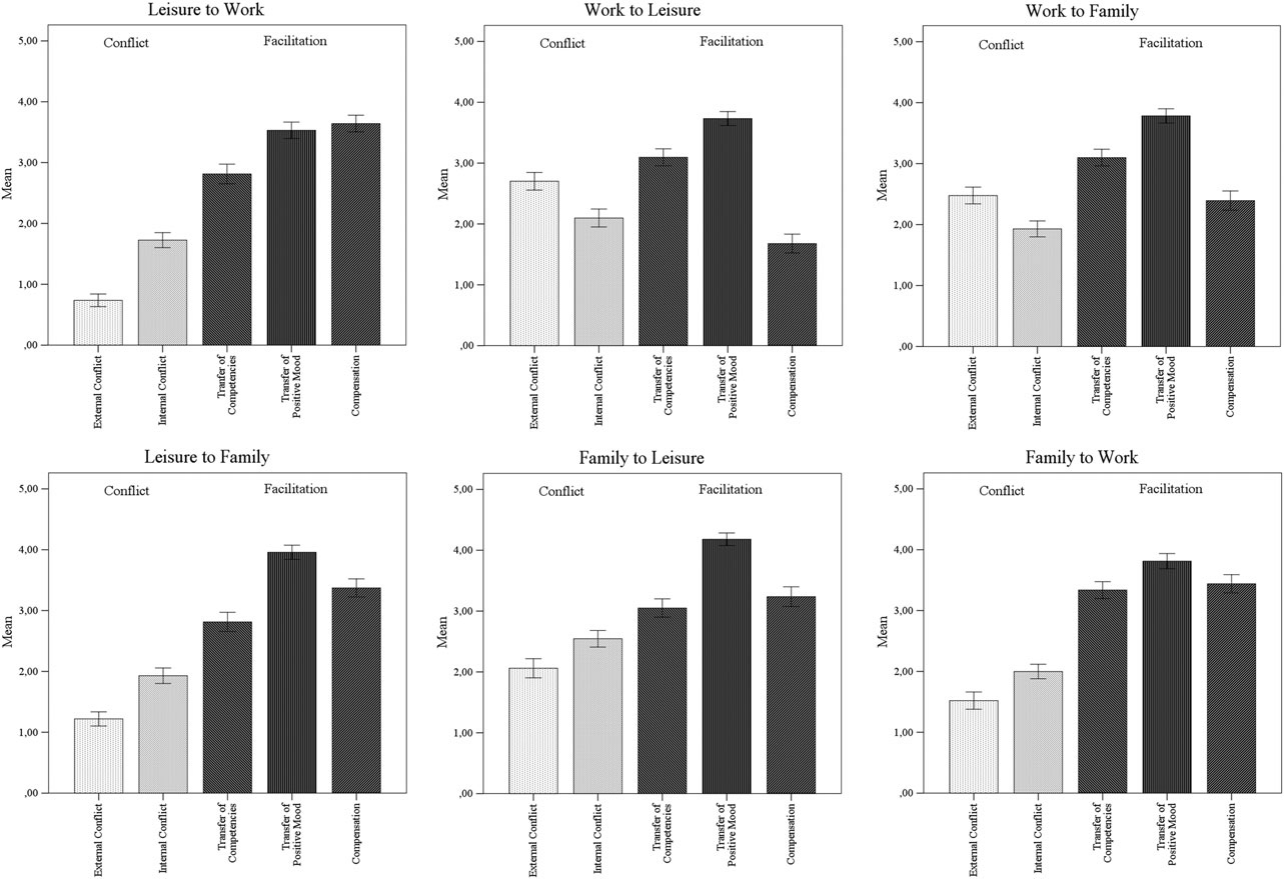
Mean values of different forms and directions of conflict and positive relations between leisure, work, and family; scales range from 0–5; error bars indicate +/−2 SE, *N* = 277

Figure 2 presents the means of the different forms of conflict. Mean values of life domain conflict differed significantly between the two forms, internal and external, and the three life domains, work, family, and leisure. Because source and recipient of conflict are intertwined in the measure and therefore cannot be tested independently, two separate multiple analyses of variance (MANOVAs) were conducted, one for life domain as source of conflict, *F*[1.85, 511.82] = 142.73, *p* < .001, *η*_p_^2^ = 0.341, and one for life domain as recipient, *F*[1.85, 510.10] = 193.77, *p* < .001, *η*_p_^2^ = 0.412. Leisure was the lowest source of conflict (*M* = 1.40, *SE* = 0.04). Note that this does not seem to be the case because leisure does not interact with the other life domains: Leisure received most conflict from the other life domains (*M* = 2.35, *SE* = 0.05). Thus, Hypotheses 1 and 2 were supported. Work, the highest source of conflict (*M* = 2.30, *SE* = 0.05), received the least conflict (*M* = 1.50, *SE* = 0.04) from the other two life domains (all *t*[276] > 5.07, all *p* < .001, *d* = |0.32–1.14|). Overall, people reported higher internal (*M* = 2.04, *SE* = 0.04) than external (*M* = 1.79, *SE* = 0.05) conflict, *F*(1, 276) = 28.24, *p* < .001, *η*_p_^2^ = 0.093. Moreover, participants reported the highest conflict for work as source (*M* = 2.30, *SE* = 0.05) followed by family (*M* = 2.03, *SE* = 0.05).

### Prevalence of different forms of facilitation

Figure 2 also presents the means of the different forms of facilitation. The degree of facilitation differed significantly between the life domains and regarding the three facets (positive transfer of competencies, positive transfer of mood, and compensation). Because the measures of source and recipient of facilitation are not independent, again, two separate MANOVAs were conducted, one for life domain as source of facilitation, *F*[1.88, 517.85] = 59.03, *p* < .001. *η*_p_^2^ = 0.176, and one for life domain as recipient of facilitation, *F*[1.86, 514.07] = 31.45, *p* < .001, *η*_p_^2^ = 0.102. Contrary to Hypothesis 3, family (*M* = 3.51, *SE* = 0.05) (and not leisure, *M* = 3.36, *SE* = 0.05) was the strongest source of facilitation and work was the weakest (*M* = 2.96, *SE* = 0.05). Work turned out to receive the most facilitation from the other life domains (*M* = 3.43, *SE* = 0.05) followed by family (*M* = 3.24, *SE* = 0.04). Leisure received the least facilitation (*M* = 3.16, *SE* = 0.04; all *t*[276] > 2.48, all *p* < .014, *d* = |0.11–0.69|). Overall, participants reported higher transfer of positive mood than transfer of competencies and compensation, *F*(2, 552) = 219.61, *p* < .001, *η*_p_^2^ = 0.442. Post hoc tests showed that there was no significant difference between transfer of competencies and compensation (*t*[276] = 1.64, *p* > .11).

### Conflict/facilitation and subjective well‐being

All forms of conflict with the leisure domain were associated with lower subjective well‐being (Table 4, column C). Thus, Hypothesis 4 was supported. In addition, as assumed in Hypothesis 5, leisure as a source as well as recipient of facilitation was associated with higher subjective well‐being (Table 5, column C).

### Stability

Both conflict and facilitation remained quite stable over a period of six months (Table 4, column A). Six‐month stability of subjective well‐being varied between *r_tt_* = .88 and .90 (*p* < .001). Conflict and facilitation from and to the leisure domain were moderately to highly stable over the study period. Six‐month stability varied between *r_tt_* = .56 and .66 (*p* < .001) for conflict with the leisure domain and between *r_tt_* = .55 and .64 (*p* < .001) for facilitation, whereas work‐family conflict (in both directions) varied between *r_tt_* = .61 and .70 (*p* < .001) and work‐family facilitation (in both directions) between *r_tt_* = .58 and .66 (*p* < .001).

### Cross‐lagged longitudinal effects

As to be expected, given the high temporal stabilities, there were only very few significant longitudinal effects for the period of six months and even fewer for the period of 12 months (Tables 4 and 5, columns D, E, F, and G). This very high stability did not allow us to address research questions, RQ1 and RQ2, which presuppose that there is some temporal variability in the variables under consideration.

## Discussion

The present study investigated the positive and negative relationships between leisure and the life domains of work and family, their temporal stabilities, and their associations with subjective well‐being. To our knowledge, this is the first longitudinal study including conflict and facilitation simultaneously in three life domains—including not only work and family but also leisure—across three measurement points spanning a total of one year.

The study has three main findings. First, leisure is a source and recipient of conflict and facilitation between both work and family. The degree of life domain conflict and facilitation varied depending on the form (internal, external for conflict and transfer of positive mood, transfer of competencies, and compensation for facilitation) and direction (source and recipient) of the inter‐domain relation. Second, conflict with the leisure domain was negatively associated and facilitation with leisure positively associated with subjective well‐being when considering cross‐sectional relations. Third, the longitudinal analyses revealed that conflict and facilitation with the leisure domain remained highly stable over the study period of one year. Because of the high stabilities of the inter‐domain relations, almost no lagged effects were detected.

### Limitations

One of the limitations of the study concerns that the data relied on self‐report from one source. Thus, we cannot rule out that associations between inter‐domain relations and subjective well‐being were partly because of the common method. However, several authors showed that common method variance is not unconditionally a major problem (e.g., Crampton & Wagner, 1994; Spector, 2006). Meade, Watson, and Kroustalis (2007) concluded that the extent among correlations biased by common method variance is likely to be small or moderate in most instances. Moreover, the constructs under investigation are inherently subjective, namely, the subjectively perceived inter‐domain relations and subjective well‐being. An external source (e.g., a colleague, the spouse, or a leisure‐related social contact) could not easily address the question of how employees experience the interplay of different life domains, and others' evaluations of a target person's feelings and experiences are not to be expected to be more reliable and valid than the target person's own reports.

Another limitation is the self‐selection of our study participants. We cannot rule out the assumption that people who experiences very high conflict between life domains would not have taken part in the study because of the lack of time.

An additional shortcoming of the present study is that it did not take into account the interactions of the life domains that might occur in everyday life. For instance, family and leisure often go hand in hand: A family visit to the museum is both family and leisure time. Similarly, asking one's spouse for advice regarding a difficult issue at work concerns the work domain as well as the spousal relationship. In fact, there might be individual differences on how people manage the boundaries between work, family, and leisure. These individual differences in boundary management might affect inter‐domain relation and subjective well‐being (e.g., Bulger, Matthews, & Hoffman, 2007; Kossek, Ruderman, Braddy, & Hannum, 2012). Moving beyond conflict and facilitation, the research on inter‐domain relations might profit from assessing the degree of integration and segmentation between different life domains (Allen, Cho, & Meier, 2014) and investigate the association of different patterns of integration and segmentation with general and domain‐specific forms of well‐being and performance.

The present study analyzed the inter‐domain relations independently of each other with regard to their impact on subjective well‐being, so as to probe into the question on which form and direction of conflict and facilitation are associated to what degree with subjective well‐being. An alternative would be to include all of these aspects of inter‐domain relations and their statistical interactions in one overall analysis. This would allow testing possible buffering effects of facilitation on the association between life domain conflict and subjective well‐being. For instance, one could test whether facilitation from leisure to the work domain might be a buffer in the negative association between work‐family conflict and well‐being. Such a complex model, however, would also require more participants than we had included in the present study to derive reliable estimates.

### Prevalence of different forms of conflict and facilitation

One of the main findings of the present study is that conflict and facilitation as well as being the source or the recipient of conflict and facilitation were not symmetrical across the three life domains. Whereas leisure was not a strong source of conflict, it turned out to receive the most conflict of the other life domains, particularly regarding external conflict. In line with previous studies, work was a stronger source of conflict than the family domain (e.g., Frone et al., 1992; Kinnunen et al., 2006). This is not surprising, because work and family demands might be experienced more often as obligations, whereas leisure activities are, by definition, voluntary and hence easier to give up than work‐related or family‐related demands. This does not, however, implies that leisure is irrelevant and should be given up in order to make one's life easier: Leisure turned out to be a strong source of facilitation for family and work. This implies that leisure provides opportunities to overcome setbacks at work or in the family.

Family was the strongest source of facilitation, and work received most facilitation from the other life domains. This finding supports the notion by Frone et al. (1992) that work and family boundaries are asymmetrically permeable.

### Inter‐domain relations and subjective well‐being

The second main result of this study is that, cross‐sectionally, conflict with the leisure domain was negatively associated and facilitation with leisure positively associated with subjective well‐being. This finding is consistent with previous results regarding work‐family conflict and facilitation. The pattern of findings concerning inter‐domain relations and subjective well‐being was largely independent of the form and direction of conflict and facilitation. Put differently, conflict hurts, and facilitation helps subjective well‐being, regardless of which life domain is the source or recipient. This result is consistent with meta‐analytic findings for correlates of conflict (Allen, Herst, Bruck, & Sutton, 2000) and facilitation (McNall et al., 2009). Although we found similar associations for all forms of conflict and subjective well‐being, this does not imply that the same mechanism drive them. It might be that the different forms of conflict (external/internal) are differentially associated with subjective well‐being. Our measure of subjective well‐being includes health‐related well‐being, namely, the self‐reported psychosomatic symptoms. Different paths are conceivable that could connect behavior/experiences with subjective well‐being and particularly with physical states. Two major ones are (1) health‐related behavior and (2) stress. In the context of life domain conflict, both paths might play a role. First, external conflict that is caused by time constraints might be associated negatively with health‐related well‐being through lower behavioral involvement in health promoting activities due to a lack of time. Second, conflict is associated with increased levels of stress (Amstad et al., 2011). Internal conflict is by definition caused by psychological involvement in thoughts and worries of one life domain while being in another life domain. They most likely lead to increased stress experiences and therefore might be negatively related to subjective well‐being. Moreover, stress is likely to be associated with unhealthy behavior such as alcohol use, unhealthy nutrition, or physical inactivity (e.g., Ng & Jeffery, 2003; Sinha & Jastreboff, 2013). It might be fruitful to analyze separately the mechanisms that link conflict and subjective well‐being for the different forms of conflict.

### Longitudinal relations

The third main result of this study is that leisure conflict and facilitation were highly stable over the course of one year, yielding comparable stability coefficients as the relations between work and family. This is surprising as people might have an easier time giving up a hobby or leisure time activities when they experience conflict with other life domains than when confronted with the more mandatory demands of the work and family domain.

The high stabilities of inter‐domain relations are plausible when taking into account the findings of a meta‐analysis by Michel, Kotrba, Mitchelson, Clark, and Baltes (2011), which showed that stable personality traits are among the strongest predictors of work‐family conflict. There is also evidence that facilitation is rooted in stable personality factors (Wayne, Musisca, & Fleeson, 2004). Thus, the stability of conflict and facilitation might reflect the stability in personality. Furthermore, the high stability of the inter‐domain relations might also be explained by people's boundary management. Allen et al. (2014) pointed out that the segmentation between the life domains work and family is associated both with conflict and facilitation between the life domains. Hecht and Allen (2009) found that boundary strength between the life domains was relatively stable over time. Moreover, Cho, Tay, Allen, and Stark (2013) showed in their study a dispositional factor for experiencing conflict as well as facilitation between the life domains in both directions (work‐to‐family and family‐to‐work). This disposition might help to explain that conflict and facilitation between leisure and the other two life domains remained stable over the course of one year in the present study.

Another source of conflict and facilitation are the resources (e.g., high income that allows to outsource household tasks and support in childcare by relatives) a person has at their disposal to deal with the demands of being engaged in multiple life domains (e.g., Grzywacz & Butler, 2005; Voydanoff, 2004). This might suggest that the factors that determine conflict and facilitation across leisure, work, and family were highly stable over the course of one year. The present study cannot disentangle these potential causes of the high stability and estimate their relative impact. Future studies addressing this issue need to include multiple measures of personality, resources, and demands, as well as conflict and facilitation over time.

One of the consequences of the high stability of inter‐domain relations was that there was no sufficient temporal variability to detect time‐lagged relations of conflict or facilitation with subjective well‐being. The association of inter‐domain relations and subjective well‐being might be stable and settled at the age between 30‐ and 55‐year‐old adults. Time‐lagged relations are probably best studied at the very beginning of a transition in one of these domains (e.g., taking on a new leisure activity, starting to work, and having a child) as then the inter‐domain relations are less stable, and effects of newly formed inter‐domain relations on later subjective well‐being (and vice versa) can be detected. Note that the lack of lagged effects in the current study do not necessarily imply that there is no causal relation between conflict/facilitation and subjective well‐being. Maybe the time lag of six months was either too long or too short to capture the impact of conflict/facilitation on subjective well‐being. The effects of inter‐goal relations on subjective well‐being might be immediate when they are experienced and evaporate after days or weeks. Day‐by‐day assessment of the constructs would allow detecting such short‐term within‐person effects. Alternatively, the associations between inter‐domain relations and subjective well‐being might have already been established at our first measurement occasion as participants have settled into routines concerning their work, family, and leisure behavior over longer periods of time and are highly stable unless circumstances change (e.g., birth of a child, divorce, and change of job).

### Theoretical implications

The current study emphasizes the importance of leisure as a significant life domain in middle adulthood. Leisure receives substantial conflict from work and family and, at the same time, is a source of facilitation for work and family. The effects of conflict with and facilitation stemming from the leisure domain on subjective well‐being are in the same direction and comparable with those stemming from work‐family relations. This suggests that the adverse effects of inter‐domain conflict and beneficial effects of inter‐domain facilitation are independent of the specific life domains that are involved and that the psychological costs and benefits are domain general. This finding stresses this importance of including leisure‐related goals into the study of goal relations.

Most importantly, the present study adds two aspects to the notion of Frone et al. (1992) that work and family are asymmetrically permeable. First, our results indicate that the boundaries between leisure and the other two life domains are also asymmetrically permeable: Leisure is to lesser degree a source of conflict than work and family, and it is to a higher degree a recipient of conflict than work and family. This suggests that leisure is more permeable for conflict than work and family. Second, permeability is different for conflict and facilitation between the life domains. Mirroring the results regarding conflict — work received less conflict than family or leisure —, work received more facilitation than family and leisure. In other words, work receives the least conflict and the most facilitation from other life domains.

### Practical implications

Engaging in leisure activities might enhance subjective well‐being in two different ways, namely, via leisure satisfaction and via facilitation of the work and the family domain (Newman et al., 2014). To invest time into leisure activities might support positive functioning in life overall. For companies, this could mean that it would be good to invest not only into career counseling of their employees but also integrate leisure activities in counseling (Tinsley & Tinsley, 2015).

The high stability of the experienced conflict and facilitation over time points out the fact that to experience conflict between life domains is for some people a constant problem in their lives. When we take into account the consistent negative association with subjective well‐being, it seems to be valuable to tailor interventions for exactly the people who experienced conflict over a long period of time. However, one could also argue that in following more preventive approach, it is also promising to implement interventions for younger organizational newcomers so that they are encouraged to develop beneficial habits of coordinating their work, family, and leisure goals and activities (see also Wiese & Knecht, 2015). All these interventions should consider the individual preferences for integration versus segmentation. Most likely, not one intervention fits all.

### Conclusion

The main contribution of the present study is the inclusion of the leisure domain. This allowed a more comprehensive assessment of the inter‐domain relations. Another strength is the three‐wave longitudinal design that allowed to assess relations over time and to detect their high stability over time.

The present study points to the positive effects of engaging in leisure activities. Leisure seems to be a major source of facilitation and only a minor source of conflict for work and family. Wiese et al. (2010) showed in their experimental work the beneficial role of inter‐domain compensation for emotional recovery after a setback. Engaging in leisure activities represents an additional source of compensation for work‐related and family‐related setbacks. Thus, we conclude that engaging in more life domains and roles is not necessarily an additional stressor but can constitute an additional opportunity for facilitation (Michel et al., 2011).

## Supporting information

Supporting info item

Supporting info item

Supporting info item

Supporting info item

Supporting info item

Supporting info item
