# Supplementary material for: Going beyond work and family: A longitudinal study on the role of leisure in the work–life interplay
Source: J Organ Behav. 2016 Mar 4;37(7):1061–77. doi: 10.1002/job.2098 (PMC6084294; doi:10.1002/job.2098)
Supplement: Supplementary file 1 — Supporting info item [file JOB-37-1061-s001.docx]

**Data Transparency Appendix**

The data reported in this manuscript were collected as part of a larger project encompassing a total of four measurement points (data of measurement point 4 is still in the process of being collected) as well as a measurement burst phase of a total of 21 days of data collection in a subsample of the longitudinal survey.

Findings from some of the cross-sectional data have been reported in MS 1 (published). MS1 focused on gender differences in the association of overall life-domain conflict and facilitation with psychosomatic complaints. MS1 does not contain any differentiation of the various facets of inter-domain relations and is based exclusively on the first measurement occasion. MS 2 (current ms) focuses on the role of leisure in the interplay of different life-domains and on the association between conflict and facilitation between the life-domains and subjective well-being over the course of one year.

As is typical for such comprehensive and time intensive longitudinal and experience sampling studies, several other manuscripts are planned. MS3 (planned) focuses on SOC-strategies and goal conflict and facilitation in everyday life. MS4 (planned) will focus on the role of domain-specific control in the interplay of life-domains and goal pursuit and will also include data from the MIDUS study (Midlife in the US). MS5 (planned) focuses on the role of probability of goal attainment and importance of goals in multiple goal pursuit. MS6 (planned) will investigate the dynamics of the prioritization within and between goal domains and their relation to goal focus, indicators of emotional well-being, and perceived control in the measurement burst part of the study combined with the prediction of prioritization from measurement point 1 and well-being outcomes at measurement points 2, 3, and 4 (after controlling for well-being at measurement point 1), as well as changes in goal variables (e.g., distance to goal, likelihood of goal achievement, etc.). MS7 (planned) will explore the association of contextual variables in the measurement burst as predictors of engagement in different life domains and long-term indicators of goal achievement (T3 and T4). MS8 (planned) will investigate the content of personal goals and their changes over time in relation to goal conflict and facilitation as well as goal importance. MS9 (planned) will investigate the degree of separation and integration of the life-domains work, family, and leisure in everyday life and its association with life-domain conflict, facilitation and wellbeing with data of the measurement burst as well as T1, T2, T3 and T4.

*Data transparency table*

| **Variables** | **Source** | Time  Point | **MS1** Status: published | **MS2** Status: Current | **MS3** Status: planned | **MS4** Status: planned | **MS5** Status: planned | **MS6** Status: planned | **MS7** Status: planned | **MS8** Status: planned | **MS9** Status: planned |
| --- | --- | --- | --- | --- | --- | --- | --- | --- | --- | --- | --- |
| **Socio-Demographic Variables** |  | T1 | X | X | X | X | X | X | X | X | X |
|  |  | T2 |  |  |  |  |  |  |  |  |  |
|  |  | T3 |  |  |  |  |  |  |  |  |  |
|  |  | T4 |  |  |  |  |  |  |  |  |  |
| **Mood** | Steyer, R., Schwenkmezger, P., Notz, P. & Eid, M. (1997). Der Mehrdimensionale Befindlichkeitsfragebogen (MDBF). *Diagnostica, 40,* 320-328. | T1 |  | X |  | X |  | X | X |  | X |
|  |  | T2 |  | X |  | X |  | X | X |  | X |
|  |  | T3 |  | X |  | X |  | X | X |  | X |
|  |  | T4 |  |  |  |  |  | X | X |  | X |
| **SOC Strategies** | Baltes, P. B., M. M. Baltes, et al. (1999). *The measurement of selection, optimization, and compensation (SOC) by self report: Technical report 1999*. Berlin, Max Planck Institute for Human Development. | T1 |  |  |  |  | X | X |  |  |  |
|  |  | T2 |  |  |  |  | X |  |  |  |  |
|  |  | T3 |  |  |  |  | X |  |  |  |  |
|  |  | T4 |  |  |  |  |  |  |  |  |  |
| **Life Satisfaction** | Diener, E., Emmons, R.T., Larsen, R.J., & Griffin, S. (1985). The Satisfaction With Life Scale*. Journal of Personality Assesment, 49*, 71-75. | T1 |  | X |  |  | X | X | X |  | X |
|  |  | T2 |  | X |  |  | X | X | X |  | X |
|  |  | T3 |  | X |  |  | X | X | X |  | X |
|  |  | T4 |  |  |  |  |  | X | X |  | X |

| **General Self-Efficacy Beliefs** | Schwarzer, R. & Jerusalem, M. (Hrsg.) (1999). *Skalen zur Erfassung von Lehrer- und Schülermerkmalen. Dokumentation der psychometrischen Verfahren im Rahmen der Wissenschaftlichen Begleitung des Modellversuchs Selbstwirksame Schulen.* Berlin: Freie Universität Berlin. | T1 |  |  |  | X |  |  |  |  |  |
| --- | --- | --- | --- | --- | --- | --- | --- | --- | --- | --- | --- |
|  |  | T2 |  |  |  |  |  |  |  |  |  |
|  |  | T3 |  |  |  |  |  |  |  |  |  |
|  |  | T4 |  |  |  |  |  |  |  |  |  |
| **Psychosomatic Symptoms** | Franke, G. H. (2002). SCL-90-R. Die Symptom-Checkliste von L.R. Derogatis. Deutsche Version. [German version of the Symptom Check List of L.R. Derogatis]. Göttingen: Beltz Test. Original: Derogatis, L. R. (1986). SLR-90-R. Self-report symptom inventory. In CIPS (Ed.), *Internationale Skalen für Psychiatrie.* Weinheim: Beltz. | T1 | X | X |  |  |  | X |  |  | X |
|  |  | T2 |  | X |  |  |  | X |  |  | X |
|  |  | T3 |  | X |  |  |  | X |  |  | X |
|  |  | T4 |  |  |  |  |  | X |  |  | X |
|  |  | MB M |  |  |  |  |  | X |  |  |  |
|  |  | MB E |  |  |  |  |  | X |  |  |  |
| **Big Five Inventory  (Short Version)** | Rammstedt, B., & John, O. P. (2005). Kurzversion des Big Five Inventory (BFI-K): Entwicklung und Validierung eines ökonomischen Inventars zur Erfassung der fünf Faktoren der Persönlichkeit. *Diagnostica, 51*, 195-206. | T1 | X^1^ |  |  |  | X |  |  |  |  |
|  |  | T2 |  |  |  |  | X |  |  |  |  |
|  |  | T3 |  |  |  |  | X |  |  |  |  |
|  |  | T4 |  |  |  |  |  |  |  |  |  |
| **Personal goals** | Ebner, N. C., Freund, A. M., & Baltes, P. B. (2006). Developmental changes in personal goal orientation from young to late adulthood: From striving for gains to maintenance and prevention of losses*. Psychology and Aging, 21*, 664-678 | T1 |  |  |  |  |  |  | X | X |  |
|  |  | T2 |  |  |  |  |  |  |  | X |  |
|  |  | T3 |  |  |  |  |  |  |  | X |  |
|  |  | T4 |  |  |  |  |  |  |  | X |  |
| **Importance of Personal Goals** | Freund, A. M., Hennecke, M., & Riediger, M. (2010). Age-related differences in outcome and process goal focus. *European Journal of Developmental Psychology*, *7*, 198-222. | T1 |  |  |  |  | X | X |  | X |  |
|  |  | T2 |  |  |  |  | X | X |  | X |  |
|  |  | T3 |  |  |  |  | X | X |  | X |  |
|  |  | T4 |  |  |  |  |  | X |  | X |  |
|  |  | MB M |  |  |  |  |  | X |  | X |  |
|  |  | MB E |  |  |  |  |  | X |  | X |  |
| **Goal Approximation** | Freund, A. M., Hennecke, M., & Riediger, M. (2010). Age-related differences in outcome and process goal focus. *European Journal of Developmental Psychology*, *7*, 198-222. | T1 |  |  |  |  |  | X |  |  |  |
|  |  | T2 |  |  |  |  |  | X |  |  |  |
|  |  | T3 |  |  |  |  |  | X |  |  |  |
|  |  | T4 |  |  |  |  |  | X |  |  |  |
|  |  | MB M |  |  |  |  |  | X |  |  |  |
|  |  | MB E |  |  |  |  |  | X |  |  |  |
| **Satisfaction with Goals** | Freund, A. M., Hennecke, M., & Riediger, M. (2010). Age-related differences in outcome and process goal focus. *European Journal of Developmental Psychology*, *7*, 198-222. | T1 |  |  |  |  |  | X |  |  |  |
|  |  | T2 |  |  |  |  |  | X |  |  |  |
|  |  | T3 |  |  |  |  |  | X |  |  |  |
|  |  | T4 |  |  |  |  |  | X |  |  |  |
|  |  | MB M |  |  |  |  |  | X |  |  |  |
|  |  | MB E |  |  |  |  |  | X |  |  |  |
| **Distance to Goal** | Freund, A. M., Hennecke, M., & Riediger, M. (2010). Age-related differences in outcome and process goal focus. *European Journal of Developmental Psychology*, *7*, 198-222. | T1 |  |  |  |  |  | X | X |  |  |
|  |  | T2 |  |  |  |  |  | X | X |  |  |
|  |  | T3 |  |  |  |  |  | X | X |  |  |
|  |  | T4 |  |  |  |  |  | X | X |  |  |
|  |  | MB M |  |  |  |  |  | X | X |  |  |
|  |  | MB E |  |  |  |  |  | X | X |  |  |
| **Probability of Goal Attainment** | Freund, A. M., Hennecke, M., & Riediger, M. (2010). Age-related differences in outcome and process goal focus. *European Journal of Developmental Psychology*, *7*, 198-222. | T1 |  |  |  |  | X | X | X |  |  |
|  |  | T2 |  |  |  |  | X | X | X |  |  |
|  |  | T3 |  |  |  |  | X | X | X |  |  |
|  |  | T4 |  |  |  |  |  | X | X |  |  |
|  |  | MB M |  |  |  |  |  | X | X |  |  |
|  |  | MB E |  |  |  |  |  | X | X |  |  |
| **Projected Goal Engagement** | Newly developed for this study | T1 |  |  |  |  |  | X | X |  |  |
|  |  | T2 |  |  |  |  |  | X | X |  |  |
|  |  | T3 |  |  |  |  |  | X | X |  |  |
|  |  | T4 |  |  |  |  |  | X | X |  |  |
| **Goal Focus** | Newly developed for this study | T1 |  |  |  |  |  | X |  |  |  |
|  |  | T2 |  |  |  |  |  | X |  |  |  |
|  |  | T3 |  |  |  |  |  | X |  |  |  |
|  |  | T4 |  |  |  |  |  | X |  |  |  |
| **Goal Conflict** | Riediger, M., & Freund, A. M. (2004). Interference and facilitation among personal goals: Differential associations with subjective well-being and persistent goal pursuit. *Personality and Social Psychology Bulletin, 30,*1511-1523 | T1 |  |  |  | X | X |  |  | X |  |
|  |  | T2 |  |  |  | X | X |  |  | X |  |
|  |  | T3 |  |  |  | X | X |  |  | X |  |
|  |  | T4 |  |  |  |  |  |  |  | X |  |

| **Goal Facilitation** | Riediger, M., & Freund, A. M. (2004). Interference and facilitation among personal goals: Differential associations with subjective well-being and persistent goal pursuit. *Personality and Social Psychology Bulletin, 30,*1511-1523 | T1 |  |  |  | X | X |  |  | X |  |
| --- | --- | --- | --- | --- | --- | --- | --- | --- | --- | --- | --- |
|  |  | T2 |  |  |  | X | X |  |  | X |  |
|  |  | T3 |  |  |  | X | X |  |  | X |  |
|  |  | T4 |  |  |  |  |  |  |  | X |  |
| **Domains-Specific Satisfaction** | Adapted from:  Giegler, H. (1985). Rasch-Skalen zur Messung von "Arbeits- und Berufszufriedenheit", "Betriebsklima" und "Arbeits- und Berufsbelastung" auf seiten der Betroffenen. *Zeitschrift* *für* *Sozialpsychologie*, 16, 13-28 | T1 |  |  |  | X |  | X | X |  |  |
|  |  | T2 |  |  |  | X |  | X | X |  |  |
|  |  | T3 |  |  |  | X |  | X | X |  |  |
|  |  | T4 |  |  |  |  |  | X | X |  |  |
|  |  | MB M |  |  |  |  |  | X | X |  |  |
|  |  | MB E |  |  |  |  |  | X | X |  |  |
| **Domain-Specific Self-Efficacy Believes** | Adapted from:  Schyns, B. & Collani, G. v. (2002). A new occupational self-efficacy scale and its relation to personality constructs and organizational variables*. European Journal of Work and Organizational Psychology, 11*, 219 – 241. | T1 |  |  |  | X |  |  |  |  |  |
|  |  | T2 |  |  |  | X |  |  |  |  |  |
|  |  | T3 |  |  |  | X |  |  |  |  |  |
|  |  | T4 |  |  |  |  |  |  |  |  |  |
| **Domain-Specific Latitude** | Adapted from: Semmer, N., Zapf, D., & Dunckel, H. (1999). Instrument zur Stressbezogenen Tätigkeitsanalyse (ISTA). In H. Dunckel (Ed.), *Handbuch psychologischer Arbeitsanalyseverfahren* (Vol. 14, pp. 179-204). Zürich: Vdf, Hochschulverlag an der ETH Zürich. | T1 |  |  |  | X |  |  | X |  |  |
|  |  | T2 |  |  |  | X |  |  | X |  |  |
|  |  | T3 |  |  |  | X |  |  | X |  |  |
|  |  | T4 |  |  |  |  |  |  | X |  |  |
| **Life-Domain Facilitation** | Adapted from:  Wiese, B.S., Seiger, C.P., Schmid, C.M., & Freund, A.M. (2010). Beyond conflict: functional facets of the work-family interplay. *Journal of Vocational Behavior, 77*, 104-117. | T1 | X | X |  | X |  |  |  |  | X |
|  |  | T2 |  | X |  | X |  |  |  |  | X |
|  |  | T3 |  | X |  | X |  |  |  |  | X |
|  |  | T4 |  |  |  |  |  |  |  |  | X |
| **Life-Domain Conflict** | Adapted from:  Carlson, D.S., & Frone, M.R. (2003). Relation of behavioral and psychological involvement to a new four-factor conceptualization of work- family interference. *Journal of Business and Psychology, 17*, 515– 535. | T1 | X | X |  | X |  |  |  |  | X |
|  |  | T2 |  | X |  | X |  |  |  |  | X |
|  |  | T3 |  | X |  | X |  |  |  |  | X |
|  |  | T4 |  |  |  |  |  |  |  |  | X |

| **Mood** | Bradley, M. M., & Lang, P., J. (1994). *Measuring emotions: the self-assessment mankin and the semantic differential.* Journal of Theoretical and Experimental Psychiatry, 25, 49-59. | MB |  |  | X | X |  | X | X |  | X |
| --- | --- | --- | --- | --- | --- | --- | --- | --- | --- | --- | --- |
| **Context (where, with whom, life-domain, thoughts)** | Riediger, M., & Freund, A. M. (2008). Me against myself: Motivational conflicts and emotional development in adulthood. *Psychology and Aging, 23*, 479-494. | MB |  |  | X |  |  |  | X |  | X |
| **Momentary goal importance** | Riediger, M., & Freund, A. M. (2008). Me against myself: Motivational conflicts and emotional development in adulthood. *Psychology and Aging, 23*, 479-494. | MB |  |  |  |  |  | X | X |  |  |
| **Momentary** **goal facilitation** | Riediger, M., & Freund, A. M. (2008). Me against myself: Motivational conflicts and emotional development in adulthood. *Psychology and Aging, 23*, 479-494. | MB |  |  | X | X |  | X |  |  | X |
| **Momentary goal conflict** | Riediger, M., & Freund, A. M. (2008). Me against myself: Motivational conflicts and emotional development in adulthood. *Psychology and Aging, 23*, 479-494. | MB |  |  | X | X |  | X |  |  | X |
| **Momentary** **SOC-behavior** | Riediger, M., & Freund, A. M. (2008). Me against myself: Motivational conflicts and emotional development in adulthood. *Psychology and Aging, 23*, 479-494. | MB |  |  | X |  |  |  | X |  |  |

*Note.* ^1^ only the dimension “neuroticism” was used; MB: Measurement Burst; MB M: Online Questionnaire in the middle of the Measurement Burst; MB E: Online Questionnaire at the end of the measurement burst;
