# Supplementary material for: Going beyond work and family: A longitudinal study on the role of leisure in the work–life interplay
Source: J Organ Behav. 2016 Mar 4;37(7):1061–77. doi: 10.1002/job.2098 (PMC6084294; doi:10.1002/job.2098)
Supplement: Supplementary file 2 — Supporting info item [file JOB-37-1061-s002.docx]

*Appendix 1:* Items life-domain conflict scale

|  | **Item wording** | **form** | **domains** |
| --- | --- | --- | --- |
| 1. | How often does you job or career interfere with your family duties (such as house keeping, grocery shopping, child care?) | external | work to family |
| 2. | How often does your job or career keep you from spending the amount of time that you would like to spend with your family / partner? | external | work to family |
| 3. | How often does your job or career interfere with your family life / partnership? | external | work to family |
| 4. | How often does you job or career interfere with your leisure activities (e.g. sports, hobbies, meeting friends)? | external | work to leisure |
| 5. | How often does your job or career keep you from spending the amount of leisure time that you would like to? | external | work to leisure |
| 6. | How often does your job or career interfere with your leisure? | external | work to leisure |
| 7. | When you are at work, how often do you think about family- / partnership-related problems? | internal | family to work |
| 8. | When you are at work, how often do you think about things you need to accomplish with your family / partner? | internal | family to work |
| 9. | When you are at work, how often do you try to arrange, schedule, or perform family- /partnership-related things? | internal | family to work |
| 10. | When you are at work, how often do you think about leisure problems? | internal | leisure to work |
| 11. | When you are at work, how often do you think about things you want to accomplish in leisure? | internal | leisure to work |
| 12. | When you are at work, how often do you try to arrange, schedule, or perform leisure-related things? | internal | leisure to work |
| 13. | How often does your family life /partnership interfere with your responsibility at work, such as getting to work on time, accomplishing daily tasks, or working overtime? | external | family to work |
| 14. | How often does your family life / partnership keep you from spending the amount of time you would like to spend on job or career-related activities? | external | family to work |
| 15. | How often does your family life / partnership interfere with your job or career? | external | family to work |
| 16. | How often does your family life / partnership interfere with your leisure activities (e.g. sports, hobbies, meet friends)? | external | family to leisure |
| 17. | How often does your family life / partnership keep you from spending the amount of leisure time you would like? | external | family to leisure |
| 18. | How often does your family life / partnership interfere with your leisure activities? | external | family to leisure |
| 19. | When you spend time with your family / partner, how often do you think about work-related problems? | internal | work to family |
| 20. | When you spend time with your family / partner, how often do you think about things you need to accomplish at work? | internal | work to family |
| 21. | When you spend time with your family / partner, how often do you try to arrange, schedule, or perform job-related activities outside of your normal work hours? | internal | work to family |
| 22. | How often do you think about leisure related problems, when you spend time with your family / partner? | internal | leisure to family |
| 23. | When you spend time with your family / partner, how often do you think about things you want to accomplish in leisure time? | internal | leisure to family |
| 24. | When you spend time with your family / partner, how often do you try to arrange, schedule, or perform leisure related things (such as organizing an event)? | internal | leisure to family |
| 25. | How often do your leisure activities interfere with your responsibility at work, such as getting to work on time, accomplishing daily tasks, or working overtime? | external | leisure to work |
| 26. | How often do your leisure activities keep you from spending the amount of time you would like to spend on job or career-related activities? | external | leisure to work |
| 27. | How often do your leisure activities interfere with your job or career? | external | leisure to work |
| 28. | How often do your leisure activities interfere with your family duties (such as house keeping, grocery shopping, child care?) | external | leisure to family |
| 29. | How often do your leisure activities keep you from spending the amount of time that you would like to spend with your family / partner? | external | leisure to family |
| 30. | How often do your leisure activities interfere with your family life / partnership? | external | leisure to family |
| 31. | During leisure, how often do you think about work-related problems? | internal | work to leisure |
| 32. | During leisure, how often do you think about things you need to accomplish at work? | internal | work to leisure |
| 33. | During leisure, how often do you try to arrange, schedule, or perform job-related activities outside of your normal work hours? | internal | work to leisure |
| 34. | During leisure, how often do you think about family- / partnership-related problems? | internal | family to leisure |
| 35. | During leisure, how often do you think about things you need to accomplish with your family / partner? | internal | family to leisure |
| 36. | During leisure, schedule, or perform family- /partnership-related things? | internal | family to leisure |
