# Supplementary material for: Going beyond work and family: A longitudinal study on the role of leisure in the work–life interplay
Source: J Organ Behav. 2016 Mar 4;37(7):1061–77. doi: 10.1002/job.2098 (PMC6084294; doi:10.1002/job.2098)
Supplement: Supplementary file 3 — Supporting info item [file JOB-37-1061-s003.docx]

*Appendix 2:* Items life-domain facilitation scale

|  | **Item wording** | **Form** | **domains** |
| --- | --- | --- | --- |
|  | The experiences acquired at work... |  |  |
| 1. | ... help me to react more flexibly to the demands of family life / partnership. | competencies | work to family |
| 2. | ... strengthen my organizational abilities in family life / partnership. | competencies | work to family |
| 3. | ... strengthen my self-confidence in my family / partnership. | competencies | work to family |
| 4. | If I feel good at work, I’m in a good mood in family life/ partnership, too. | mood | work to family |
| 5. | If I feel energetic at work. I’ve got a lot of energy in my family / partnership, too. | mood | work to family |
| 6. | If I’m calm at work, I feel relaxed in my family life / partnership, too. | mood | work to family |
| 7. | If things aren’t going well at work, my family life / partnership gives me a boost. | compensation | family to work |
| 8. | After a stressful day at work, I get recharged in my family / partnership. | compensation | family to work |
| 9. | When I’m heavily burdened at work, my family / partner help me relax. | compensation | family to work |
|  | The experiences acquired in my family life / partnership ... |  |  |
| 10. | ... help me to react more flexibly to the demands at work. | competencies | family to work |
| 11. | ... strengthen my organizational abilities at work. | competencies | family to work |
| 12. | ... strengthen my self-confidence at work. | competencies | family to work |
| 13. | If I feel good in my family life / partnership, I am in a good mood at work, too. | mood | family to work |
| 14. | If I feel energetic in my family life /partnership, I’ve got a lot of energy at work as well. | mood | family to work |
| 15. | If I am clam in my family life / partnership, I feel relaxed at work, too. | mood | family to work |
| 16. | If things aren’t going well in my family life / partnership, my job gives my a boost. | compensation | work to family |
| 17. | If things are stressful in my family life / partnership, I get recharged at work. | compensation | work to family |
| 18. | When I’m heavily burdened in my family life / partnership (e.g. conflicts, illness), my job helps me relax. | compensation | work to family |
|  | The experiences acquired at work... |  |  |
| 19. | ... help me to react more flexibly to the demands of my leisure. | competencies | work to leisure |
| 20. | ... strengthen my organizational abilities in leisure, | competencies | work to leisure |
| 21. | ... strengthen my self-confidence leisure. | competencies | work to leisure |
| 22. | If I feel good at work, I’m in a good mood in leisure, too. | mood | work to leisure |
| 23. | If I feel energetic at work. I’ve got a lot of energy for my leisure activities, too. | mood | work to leisure |
| 24. | If I’m calm at work, I feel relaxed in leisure, too. | mood | work to leisure |
| 25. | If things aren’t going well at work, my leisure gives me a boost. | compensation | leisure to work |
| 26. | After a stressful day at work, I get recharged in leisure. | compensation | leisure to work |
| 27. | When I’m heavily burdened at work, my leisure helps me relax. | compensation | leisure to work |
|  | The experiences acquired from leisure... |  |  |
| 28. | ... help me to react more flexibly to the demands at work. | competencies | leisure to work |
| 29. | ... strengthen my organizational abilities at work. | competencies | leisure to work |
| 30. | ... strengthen my self-confidence at work. | competencies | leisure to work |
| 31. | If I feel good in leisure, I am in a good mood at work, too. | mood | leisure to work |
| 32. | If I feel energetic in my leisure, I’ve got a lot of energy at work as well. | mood | leisure to work |
| 33. | If I am calm in my leisure, I feel relaxed at work, too. | mood | leisure to work |
| 34. | If things aren’t going well in my leisure, my job gives my a boost. | compensation | work to leisure |
| 35. | If my leisure activities (sports, travelling, organizing events or else) are overburdening, I get recharged at work. | compensation | work to leisure |
| 36. | If there is much going on in leisure (a lot to organize, sports, parties etc.), my work helps my to detach and to relax. | compensation | work to leisure |
|  | The experiences acquired from leisure... |  |  |
| 37. | ... help me to react more flexibly to the demands of family life / partnership. | competencies | leisure to family |
| 38. | ... strengthen my organizational abilities in family life / partnership. | competencies | leisure to family |
| 39. | ... strengthen my self-confidence in my family / partnership. | competencies | leisure to family |
| 40. | If I feel good in leisure, I am in a good mood in family life / partnership, too. | mood | leisure to family |
| 41. | If I feel energetic in my leisure, I’ve got a lot of energy in my family / partnership as well. | mood | leisure to family |
| 42. | If I am clam in my leisure, I feel relaxed in family life / partnership, too. | mood | leisure to family |
| 43. | If things aren’t going well in my leisure, my family life / partnership gives me boost. | compensation | family to leisure |
| 44. | If my leisure activities (sports, travelling, organizing events or else) are overburdening, I get recharged in my family / partnership. | compensation | family to leisure |
| 45. | When I’m heavily burdened in my family life / partnership (e.g. conflicts, illness), my family / partner help me relax. | compensation | family to leisure |
|  | The experiences acquired in my family life / partnership ... |  |  |
| 46. | ... help me to react more flexibly to the demands of my leisure. | competencies | family to leisure |
| 47. | ... strengthen my organizational abilities in leisure, | competencies | family to leisure |
| 48. | ... strengthen my self-confidence leisure. | competencies | family to leisure |
| 49. | If I feel good in my family life / partnership, I am in a good mood in leisure, too. | mood | family to leisure |
| 50. | If I feel energetic in my family life /partnership, I’ve got a lot of energy for my leisure activities. | mood | family to leisure |
| 51. | If I am clam in my family life / partnership, I feel relaxed in leisure, too. | mood | family to leisure |
| 52. | If things aren’t going well in my family life / partnership, my leisure gives my a boost. | compensation | leisure to family |
| 53. | If things are stressful in my family life / partnership, I get recharged in leisure. | compensation | leisure to family |
| 54. | When I’m heavily burdened in my family life / partnership (e.g. conflicts, illness), my leisure helps me relax | compensation | leisure to family |
