# Supplementary material for: Going beyond work and family: A longitudinal study on the role of leisure in the work–life interplay
Source: J Organ Behav. 2016 Mar 4;37(7):1061–77. doi: 10.1002/job.2098 (PMC6084294; doi:10.1002/job.2098)
Supplement: Supplementary file 5 — Supporting info item [file JOB-37-1061-s005.docx]

| *Appendix 4:* Mean values (M), Standard deviations (SD), correlations and Cronbach alphas (in parenteses) of all conflict and facilitation subscales at T2 | | | | | | | | | | | | | | | | | | | | | | | | | | | | | | | | |
| --- | --- | --- | --- | --- | --- | --- | --- | --- | --- | --- | --- | --- | --- | --- | --- | --- | --- | --- | --- | --- | --- | --- | --- | --- | --- | --- | --- | --- | --- | --- | --- | --- |
|  | M | SD | 1 | 2 | 3 | 4 | 5 | 6 | 7 | 8 | 9 | 10 | 11 | 12 | 13 | 14 | 15 | 16 | 17 | 18 | 19 | 20 | 21 | 22 | 23 | 24 | 25 | 26 | 27 | 28 | 29 | 30 |
| **Leisure to Work** |  |  |  |  |  |  |  |  |  |  |  |  |  |  |  |  |  |  |  |  |  |  |  |  |  |  |  |  |  |  |  |  |
| 1. External conflict | 0.74 | 0.87 | (.85) |  |  |  |  |  |  |  |  |  |  |  |  |  |  |  |  |  |  |  |  |  |  |  |  |  |  |  |  |  |
| 2. Internal conflict | 1.73 | 1.03 | .22^**^ | (.86) |  |  |  |  |  |  |  |  |  |  |  |  |  |  |  |  |  |  |  |  |  |  |  |  |  |  |  |  |
| 3. Transfer of Competencies | 2.81 | 1.35 | .16^**^ | 0.10 | (.89) |  |  |  |  |  |  |  |  |  |  |  |  |  |  |  |  |  |  |  |  |  |  |  |  |  |  |  |
| 4. Transfer of positive Mood | 3.53 | 1.13 | 0.06 | -0.03 | .45^**^ | (.90) |  |  |  |  |  |  |  |  |  |  |  |  |  |  |  |  |  |  |  |  |  |  |  |  |  |  |
| 5. Compen-sation | 3.64 | 1.14 | .15^*^ | 0.10 | .36^**^ | .29^**^ | (.91) |  |  |  |  |  |  |  |  |  |  |  |  |  |  |  |  |  |  |  |  |  |  |  |  |  |
| **Leisure to Family** |  |  |  |  |  |  |  |  |  |  |  |  |  |  |  |  |  |  |  |  |  |  |  |  |  |  |  |  |  |  |  |  |
| 6. External conflict | 1.22 | 0.97 | .57^**^ | .13^*^ | 0.12 | 0.04 | .13^*^ | (.83) |  |  |  |  |  |  |  |  |  |  |  |  |  |  |  |  |  |  |  |  |  |  |  |  |
| 7. Internal conflict | 1.93 | 1.06 | .28^**^ | .44^**^ | 0.09 | -0.03 | 0.04 | .28^**^ | (.81) |  |  |  |  |  |  |  |  |  |  |  |  |  |  |  |  |  |  |  |  |  |  |  |
| 8. Transfer of Competencies | 2.81 | 1.31 | .19^**^ | 0.09 | .72^**^ | .41^**^ | .29^**^ | .14^*^ | 0.08 | (.91) |  |  |  |  |  |  |  |  |  |  |  |  |  |  |  |  |  |  |  |  |  |  |
| 9. Transfer of positive Mood | 3.96 | 0.97 | 0.02 | 0.00 | .36^**^ | .60^**^ | .19^**^ | 0.05 | 0.06 | .43^**^ | (.91) |  |  |  |  |  |  |  |  |  |  |  |  |  |  |  |  |  |  |  |  |  |
| 10. Compen-sation | 3.37 | 1.24 | .14^*^ | 0.08 | .38^**^ | .24^**^ | .43^**^ | .15^*^ | .17^**^ | .37^**^ | .23^**^ | (.91) |  |  |  |  |  |  |  |  |  |  |  |  |  |  |  |  |  |  |  |  |
| **Work to Leisure** |  |  |  |  |  |  |  |  |  |  |  |  |  |  |  |  |  |  |  |  |  |  |  |  |  |  |  |  |  |  |  |  |
| 11. External conflict | 2.70 | 1.20 | .15^*^ | .12^*^ | -0.04 | -0.03 | -0.08 | .16^**^ | .18^**^ | -0.01 | 0.03 | -0.02 | (.91) |  |  |  |  |  |  |  |  |  |  |  |  |  |  |  |  |  |  |  |
| 12. Internal conflict | 2.10 | 1.23 | 0.08 | -0.09 | -0.09 | -0.03 | -.14^*^ | 0.00 | 0.05 | -0.05 | 0.01 | -.16^**^ | .24^**^ | (.90) |  |  |  |  |  |  |  |  |  |  |  |  |  |  |  |  |  |  |
| 13. Transfer of Competencies | 3.10 | 1.16 | 0.11 | 0.00 | .31^**^ | .15^*^ | .27^**^ | 0.02 | 0.02 | .32^**^ | 0.11 | .16^**^ | -0.11 | 0.07 | (.82) |  |  |  |  |  |  |  |  |  |  |  |  |  |  |  |  |  |
| 14 Transfer of positive Mood | 3.73 | 0.96 | -0.01 | 0.01 | 0.10 | .24^**^ | .20^**^ | 0.01 | -0.01 | .16^**^ | .26^**^ | .15^*^ | -0.08 | 0.12 | .43^**^ | (.84) |  |  |  |  |  |  |  |  |  |  |  |  |  |  |  |  |
| 15. Compen-sation | 1.68 | 1.29 | .12^*^ | -0.05 | .21^**^ | .28^**^ | 0.08 | 0.05 | 0.01 | .22^**^ | .13^*^ | 0.05 | -0.09 | .28^**^ | .28^**^ | .24^**^ | (.87) |  |  |  |  |  |  |  |  |  |  |  |  |  |  |  |
| **Family to Leisure** |  |  |  |  |  |  |  |  |  |  |  |  |  |  |  |  |  |  |  |  |  |  |  |  |  |  |  |  |  |  |  |  |
| 16. External conflict | 2.06 | 1.31 | .18^**^ | .14^*^ | -.21^**^ | -0.11 | -.15^*^ | .21^**^ | .23^**^ | -.13^*^ | -.13^*^ | -0.02 | .41^**^ | 0.06 | -0.09 | -0.09 | -0.09 | (.91) |  |  |  |  |  |  |  |  |  |  |  |  |  |  |
| 17. Internal conflict | 2.55 | 1.13 | 0.10 | .19^**^ | -0.10 | 0.10 | -.13^*^ | .17^**^ | .33^**^ | 0.02 | .12^*^ | -0.03 | .22^**^ | .51^**^ | 0.00 | 0.02 | .17^**^ | .20^**^ | (.85) |  |  |  |  |  |  |  |  |  |  |  |  |  |
| 18. Tranfer of Competencies | 3.05 | 1.25 | 0.09 | 0.12 | .48^**^ | .21^**^ | .29^**^ | 0.05 | .13^*^ | .55^**^ | .27^**^ | .26^**^ | 0.05 | -0.02 | .40^**^ | .22^**^ | .17^**^ | -.16^**^ | 0.03 | (.90) |  |  |  |  |  |  |  |  |  |  |  |  |
| 19. Transfer of positive Mood | 4.18 | 0.86 | -0.09 | 0.01 | .26^**^ | .38^**^ | .19^**^ | -0.11 | 0.00 | .30^**^ | .46^**^ | .22^**^ | 0.00 | -0.02 | .17^**^ | .42^**^ | .15^*^ | -.16^**^ | 0.04 | .39^**^ | (.89) |  |  |  |  |  |  |  |  |  |  |  |
| 20. Compen-sation | 3.24 | 1.36 | 0.00 | 0.05 | .29^**^ | .32^**^ | .31^**^ | 0.05 | 0.02 | .31^**^ | .46^**^ | .22^**^ | -0.02 | 0.06 | 0.06 | .18^**^ | .20^**^ | -.41^**^ | 0.10 | .38^**^ | .33^**^ | (.93) |  |  |  |  |  |  |  |  |  |  |
| **Work to Family** |  |  |  |  |  |  |  |  |  |  |  |  |  |  |  |  |  |  |  |  |  |  |  |  |  |  |  |  |  |  |  |  |
| 21. External conflict | 2.48 | 1.15 | .14^*^ | 0.08 | -0.06 | -0.04 | -.17^**^ | .18^**^ | .16^**^ | -0.02 | 0.00 | -.13^*^ | .76^**^ | .30^**^ | -0.11 | -0.04 | -0.01 | .33^**^ | .25^**^ | 0.04 | -0.03 | -0.02 | (.85) |  |  |  |  |  |  |  |  |  |
| 22. Internal conflict | 1.93 | 1.09 | 0.07 | 0.01 | -0.07 | -0.07 | -.16^**^ | 0.02 | .16^**^ | 0.02 | -0.03 | -.19^**^ | .29^**^ | .66^**^ | 0.09 | .12^*^ | .23^**^ | 0.10 | .29^**^ | 0.04 | 0.02 | -0.05 | .33^**^ | (.89) |  |  |  |  |  |  |  |  |
| 23. Transfer of Competencies | 3.10 | 1.14 | 0.06 | -0.09 | .21^**^ | .16^**^ | .20^**^ | -0.07 | -0.06 | .18^**^ | 0.06 | 0.10 | -0.12 | 0.07 | .74^**^ | .33^**^ | .28^**^ | -0.09 | 0.01 | .26^**^ | 0.08 | 0.01 | -.14^*^ | 0.08 | (.77) |  |  |  |  |  |  |  |
| 24. Transfer of positive Mood | 3.78 | 0.97 | -0.01 | -0.02 | 0.09 | .25^**^ | 0.08 | 0.04 | -0.07 | .15^*^ | .34^**^ | 0.06 | -0.06 | 0.07 | .38^**^ | .74^**^ | .14^*^ | -.14^*^ | 0.06 | .19^**^ | .34^**^ | .25^**^ | -0.06 | 0.10 | .33^**^ | (.86) |  |  |  |  |  |  |
| 25. Compen-sation | 2.39 | 1.31 | 0.00 | -.17^**^ | .14^*^ | .18^**^ | .13^*^ | -0.05 | -.13^*^ | 0.10 | -0.01 | .15^*^ | -.12^*^ | .16^**^ | .43^**^ | .26^**^ | .64^**^ | 0.00 | 0.03 | .13^*^ | 0.08 | -0.03 | -0.10 | .17^**^ | .41^**^ | .14^*^ | (.89) |  |  |  |  |  |
| **Family to Work** |  |  |  |  |  |  |  |  |  |  |  |  |  |  |  |  |  |  |  |  |  |  |  |  |  |  |  |  |  |  |  |  |
| 26. External conflict | 1.52 | 1.18 | .24^**^ | .15^*^ | -.12^*^ | -0.08 | -0.09 | .14^*^ | .13^*^ | -0.02 | -0.10 | -0.01 | .37^**^ | .13^*^ | -0.04 | -0.04 | -0.05 | .64^**^ | .19^**^ | -0.01 | -0.08 | -.24^**^ | .35^**^ | .15^*^ | -0.02 | -0.05 | 0.00 | (.87) |  |  |  |  |
| 27. Internal conflict | 2.00 | 0.99 | .13^*^ | .67^**^ | -0.01 | -0.03 | -0.05 | 0.04 | .30^**^ | 0.01 | 0.00 | -0.02 | .14^*^ | 0.07 | -0.03 | 0.02 | 0.03 | .20^**^ | .34^**^ | 0.06 | 0.02 | 0.00 | .13^*^ | .22^**^ | -0.10 | 0.04 | -0.07 | .25^**^ | (.82) |  |  |  |
| 28. Transfer of Competencies | 3.34 | 1.16 | 0.01 | 0.01 | .36^**^ | .29^**^ | .28^**^ | -.12^*^ | -0.06 | .32^**^ | .31^**^ | .14^*^ | 0.08 | -0.02 | .20^**^ | .18^**^ | .20^**^ | -.15^*^ | 0.03 | .54^**^ | .31^**^ | .31^**^ | 0.09 | -0.01 | .18^**^ | .17^**^ | .16^**^ | -0.02 | 0.08 | (.83) |  |  |
| 29. Transfer of positive Mood | 3.81 | 1.04 | -0.03 | -0.06 | .25^**^ | .58^**^ | .20^**^ | -0.08 | -0.07 | .20^**^ | .38^**^ | .15^*^ | -0.01 | 0.06 | .18^**^ | .37^**^ | .27^**^ | -0.11 | .13^*^ | .24^**^ | .55^**^ | .31^**^ | -0.02 | -0.02 | .18^**^ | .34^**^ | .23^**^ | -0.07 | 0.09 | .38^**^ | (.87) |  |
| 30. Compen-sation | 3.44 | 1.25 | -0.08 | -0.06 | .15^*^ | .16^**^ | .37^**^ | -0.04 | -.15^*^ | 0.08 | .24^**^ | 0.00 | -0.09 | 0.03 | .19^**^ | .21^**^ | .12^*^ | -.40^**^ | -.12^*^ | .26^**^ | .29^**^ | .64^**^ | -0.10 | -0.04 | 0.11 | .25^**^ | 0.04 | -.27^**^ | -0.10 | .30^**^ | .30^**^ | (.92) |
| Note. * p < .05, ** p < .01; *N* = 277 | | | | | | | | | | | | | | | | | | | | | | | | | | | | | | | | |
