# Supplementary material for: Going beyond work and family: A longitudinal study on the role of leisure in the work–life interplay
Source: J Organ Behav. 2016 Mar 4;37(7):1061–77. doi: 10.1002/job.2098 (PMC6084294; doi:10.1002/job.2098)
Supplement: Supplementary file 6 — Supporting info item [file JOB-37-1061-s006.docx]

| *Appendix 5:* Mean values (M), Standard deviations (SD), correlations and Cronbach alphas (in parenteses) of all conflict and facilitation subscales at T3 | | | | | | | | | | | | | | | | | | | | | | | | | | | | | | | | | | | | | | | | | | | | | | | | | | | | | | | | | | | | | | |
| --- | --- | --- | --- | --- | --- | --- | --- | --- | --- | --- | --- | --- | --- | --- | --- | --- | --- | --- | --- | --- | --- | --- | --- | --- | --- | --- | --- | --- | --- | --- | --- | --- | --- | --- | --- | --- | --- | --- | --- | --- | --- | --- | --- | --- | --- | --- | --- | --- | --- | --- | --- | --- | --- | --- | --- | --- | --- | --- | --- | --- | --- | --- |
|  | M | SD | 1 | 2 | | 3 | | 4 | | 5 | | 6 | | 7 | | 8 | | 9 | | 10 | | 11 | | 12 | | 13 | | 14 | | 15 | | 16 | | 17 | | 18 | | 19 | | 20 | | 21 | | 22 | | 23 | | 24 | | 25 | | 26 | | 27 | | 28 | | 29 | | 30 | |  |
| **Leisure to Work** |  |  |  | |  | |  | |  | |  | |  | |  | |  | |  | |  | |  | |  | |  | |  | |  | |  | |  | |  | |  | |  | |  | |  | |  | |  | |  | |  | |  | |  | |  | |  | |
| 1. External conflict | 0.83 | 0.87 | (.87) | |  | |  | |  | |  | |  | |  | |  | |  | |  | |  | |  | |  | |  | |  | |  | |  | |  | |  | |  | |  | |  | |  | |  | |  | |  | |  | |  | |  | |  | |
| 2. Internal conflict | 1.64 | 1.06 | .36^**^ | | (.90) | |  | |  | |  | |  | |  | |  | |  | |  | |  | |  | |  | |  | |  | |  | |  | |  | |  | |  | |  | |  | |  | |  | |  | |  | |  | |  | |  | |  | |
| 3. Transfer of Competencies | 2.84 | 1.27 | .17^**^ | | .18^**^ | | (.91) | |  | |  | |  | |  | |  | |  | |  | |  | |  | |  | |  | |  | |  | |  | |  | |  | |  | |  | |  | |  | |  | |  | |  | |  | |  | |  | |  | |
| 4. Transfer of positive Mood | 3.51 | 1.02 | -0.06 | | 0.01 | | .49^**^ | | (.89) | |  | |  | |  | |  | |  | |  | |  | |  | |  | |  | |  | |  | |  | |  | |  | |  | |  | |  | |  | |  | |  | |  | |  | |  | |  | |  | |
| 5. Compen-sation | 3.68 | 1.13 | .13^*^ | | 0.12 | | .44^**^ | | .35^**^ | | (.91) | |  | |  | |  | |  | |  | |  | |  | |  | |  | |  | |  | |  | |  | |  | |  | |  | |  | |  | |  | |  | |  | |  | |  | |  | |  | |
| **Leisure to Family** |  |  |  | |  | |  | |  | |  | |  | |  | |  | |  | |  | |  | |  | |  | |  | |  | |  | |  | |  | |  | |  | |  | |  | |  | |  | |  | |  | |  | |  | |  | |  | |
| 6. External conflict | 1.20 | 0.88 | .65^**^ | | .22^**^ | | .15^*^ | | -0.03 | | 0.08 | | (.84) | |  | |  | |  | |  | |  | |  | |  | |  | |  | |  | |  | |  | |  | |  | |  | |  | |  | |  | |  | |  | |  | |  | |  | |  | |
| 7. Internal conflict | 1.89 | 0.97 | .32^**^ | | .34^**^ | | .20^**^ | | -0.02 | | 0.12 | | .34^**^ | | (.83) | |  | |  | |  | |  | |  | |  | |  | |  | |  | |  | |  | |  | |  | |  | |  | |  | |  | |  | |  | |  | |  | |  | |  | |
| 8. Transfer of Competencies | 2.82 | 1.22 | 0.08 | | 0.12 | | .72^**^ | | .51^**^ | | .37^**^ | | 0.07 | | .22^**^ | | (.91) | |  | |  | |  | |  | |  | |  | |  | |  | |  | |  | |  | |  | |  | |  | |  | |  | |  | |  | |  | |  | |  | |  | |
| 9. Transfer of positive Mood | 3.82 | 0.99 | -0.11 | | -0.07 | | .36^**^ | | .59^**^ | | .34^**^ | | -0.09 | | 0.09 | | .48^**^ | | (.93) | |  | |  | |  | |  | |  | |  | |  | |  | |  | |  | |  | |  | |  | |  | |  | |  | |  | |  | |  | |  | |  | |
| 10. Compen-sation | 3.31 | 1.30 | .13^*^ | | .17^**^ | | .41^**^ | | .34^**^ | | .67^**^ | | 0.10 | | .22^**^ | | .42^**^ | | .35^**^ | | (.94) | |  | |  | |  | |  | |  | |  | |  | |  | |  | |  | |  | |  | |  | |  | |  | |  | |  | |  | |  | |  | |
| **Work to Leisure** |  |  |  | |  | |  | |  | |  | |  | |  | |  | |  | |  | |  | |  | |  | |  | |  | |  | |  | |  | |  | |  | |  | |  | |  | |  | |  | |  | |  | |  | |  | |  | |
| 11. External conflict | 2.53 | 1.28 | .26^**^ | | .15^*^ | | -.13^*^ | | -0.09 | | -0.12 | | .31^**^ | | .24^**^ | | -0.12 | | -.14^*^ | | -0.06 | | (.93) | |  | |  | |  | |  | |  | |  | |  | |  | |  | |  | |  | |  | |  | |  | |  | |  | |  | |  | |  | |
| 12. Internal conflict | 2.02 | 1.18 | 0.12 | | .13^*^ | | -0.08 | | -0.01 | | -.13^*^ | | 0.10 | | .20^**^ | | -.20^**^ | | -0.09 | | -0.12 | | .25^**^ | | (.91) | |  | |  | |  | |  | |  | |  | |  | |  | |  | |  | |  | |  | |  | |  | |  | |  | |  | |  | |
| 13. Transfer of Competencies | 3.06 | 1.28 | .19^**^ | | -0.01 | | .43^**^ | | .40^**^ | | .38^**^ | | 0.12 | | 0.06 | | .36^**^ | | .28^**^ | | .37^**^ | | -0.06 | | 0.00 | | (.91) | |  | |  | |  | |  | |  | |  | |  | |  | |  | |  | |  | |  | |  | |  | |  | |  | |  | |
| 14 Transfer of positive Mood | 3.76 | 0.88 | -0.04 | | -0.06 | | .16^*^ | | .42^**^ | | .29^**^ | | -.15^*^ | | -0.07 | | .28^**^ | | .34^**^ | | .23^**^ | | -0.01 | | -0.02 | | .37^**^ | | (.87) | |  | |  | |  | |  | |  | |  | |  | |  | |  | |  | |  | |  | |  | |  | |  | |  | |
| 15. Compen-sation | 1.96 | 1.27 | 0.06 | | 0.03 | | .25^**^ | | .33^**^ | | 0.10 | | -0.05 | | 0.00 | | .22^**^ | | 0.02 | | .17^**^ | | -0.12 | | .23^**^ | | .34^**^ | | .23^**^ | | (.87) | |  | |  | |  | |  | |  | |  | |  | |  | |  | |  | |  | |  | |  | |  | |  | |
| **Family to Leisure** |  |  |  | |  | |  | |  | |  | |  | |  | |  | |  | |  | |  | |  | |  | |  | |  | |  | |  | |  | |  | |  | |  | |  | |  | |  | |  | |  | |  | |  | |  | |  | |
| 16. External conflict | 2.01 | 1.28 | 0.07 | | 0.06 | | -.21^**^ | | -.15^*^ | | -.26^**^ | | .23^**^ | | 0.11 | | -.20^**^ | | -.17^**^ | | -0.06 | | .39^**^ | | 0.04 | | -.13^*^ | | -0.09 | | -0.08 | | (.92) | |  | |  | |  | |  | |  | |  | |  | |  | |  | |  | |  | |  | |  | |  | |
| 17. Internal conflict | 2.36 | 1.07 | 0.07 | | .30^**^ | | 0.02 | | 0.08 | | 0.01 | | .16^*^ | | .39^**^ | | 0.03 | | 0.11 | | 0.04 | | .28^**^ | | .53^**^ | | -0.09 | | -0.09 | | 0.08 | | 0.12 | | (.83) | |  | |  | |  | |  | |  | |  | |  | |  | |  | |  | |  | |  | |  | |
| 18. Tranfer of Competencies | 2.99 | 1.19 | 0.11 | | 0.10 | | .52^**^ | | .38^**^ | | .37^**^ | | 0.11 | | 0.06 | | .54^**^ | | .35^**^ | | .37^**^ | | -0.07 | | -0.02 | | .43^**^ | | .33^**^ | | .26^**^ | | -.15^*^ | | -0.01 | | (.91) | |  | |  | |  | |  | |  | |  | |  | |  | |  | |  | |  | |  | |
| 19. Transfer of positive Mood | 4.04 | 0.92 | -0.09 | | -0.10 | | .17^**^ | | .42^**^ | | .32^**^ | | -0.10 | | 0.01 | | .28^**^ | | .61^**^ | | .34^**^ | | -0.05 | | -0.01 | | .23^**^ | | .35^**^ | | 0.04 | | -.17^**^ | | 0.08 | | .37^**^ | | (.94) | |  | |  | |  | |  | |  | |  | |  | |  | |  | |  | |  | |
| 20. Compen-sation | 3.17 | 1.37 | 0.08 | | 0.04 | | .34^**^ | | .31^**^ | | .43^**^ | | 0.04 | | 0.11 | | .39^**^ | | .48^**^ | | .38^**^ | | -0.05 | | 0.01 | | .26^**^ | | .17^**^ | | .13^*^ | | -.28^**^ | | 0.11 | | .46^**^ | | .43^**^ | | (.93) | |  | |  | |  | |  | |  | |  | |  | |  | |  | |  | |
| **Work to Family** |  |  |  | |  | |  | |  | |  | |  | |  | |  | |  | |  | |  | |  | |  | |  | |  | |  | |  | |  | |  | |  | |  | |  | |  | |  | |  | |  | |  | |  | |  | |  | |
| 21. External conflict | 2.43 | 1.17 | .22^**^ | | 0.10 | | -.21^**^ | | -0.10 | | -.22^**^ | | .31^**^ | | .23^**^ | | -.21^**^ | | -.18^**^ | | -.13^*^ | | .83^**^ | | .30^**^ | | -.13^*^ | | -0.04 | | -0.05 | | .41^**^ | | .33^**^ | | -.14^*^ | | -0.06 | | -0.10 | | (.88) | |  | |  | |  | |  | |  | |  | |  | |  | |  | |
| 22. Internal conflict | 1.85 | 0.97 | 0.12 | | 0.11 | | -0.12 | | -0.09 | | -.19^**^ | | 0.09 | | .33^**^ | | -.13^*^ | | -0.03 | | -0.10 | | .32^**^ | | .65^**^ | | 0.05 | | -0.05 | | .14^*^ | | 0.12 | | .34^**^ | | -0.03 | | 0.02 | | 0.02 | | .31^**^ | | (.87) | |  | |  | |  | |  | |  | |  | |  | |  | |
| 23. Transfer of Competencies | 3.18 | 1.14 | 0.06 | | -0.05 | | .33^**^ | | .31^**^ | | .22^**^ | | 0.06 | | -0.01 | | .23^**^ | | .16^*^ | | .20^**^ | | 0.04 | | -0.02 | | .65^**^ | | .33^**^ | | .37^**^ | | 0.03 | | -0.07 | | .30^**^ | | 0.12 | | 0.08 | | 0.00 | | 0.04 | | (.87) | |  | |  | |  | |  | |  | |  | |  | |
| 24. Transfer of positive Mood | 3.81 | 0.86 | -0.01 | | -.16^*^ | | 0.12 | | .40^**^ | | .21^**^ | | -0.08 | | -0.08 | | .23^**^ | | .44^**^ | | .27^**^ | | -0.02 | | -0.05 | | .39^**^ | | .68^**^ | | .15^*^ | | -.15^*^ | | -0.04 | | .27^**^ | | .41^**^ | | .28^**^ | | -0.03 | | 0.00 | | .21^**^ | | (.84) | |  | |  | |  | |  | |  | |  | |
| 25. Compen-sation | 2.57 | 1.28 | 0.12 | | -0.08 | | .18^**^ | | .21^**^ | | .18^**^ | | -0.03 | | -0.05 | | 0.12 | | 0.07 | | .21^**^ | | -0.07 | | 0.00 | | .42^**^ | | .23^**^ | | .54^**^ | | -0.03 | | -0.11 | | .22^**^ | | .19^**^ | | 0.00 | | -0.07 | | 0.08 | | .43^**^ | | .24^**^ | | (.91) | |  | |  | |  | |  | |  | |
| **Family to Work** |  |  |  | |  | |  | |  | |  | |  | |  | |  | |  | |  | |  | |  | |  | |  | |  | |  | |  | |  | |  | |  | |  | |  | |  | |  | |  | |  | |  | |  | |  | |  | |
| 26. External conflict | 1.53 | 1.13 | .24^**^ | | .18^**^ | | -.15^*^ | | -.13^*^ | | -.23^**^ | | .18^**^ | | .17^**^ | | -.17^**^ | | -.26^**^ | | -0.09 | | .32^**^ | | .24^**^ | | -0.10 | | -0.03 | | 0.11 | | .62^**^ | | .23^**^ | | -0.07 | | -0.10 | | -.21^**^ | | .43^**^ | | .22^**^ | | 0.02 | | -0.12 | | 0.05 | | (.89) | |  | |  | |  | |  | |
| 27. Internal conflict | 2.00 | 1.03 | .18^**^ | | .72^**^ | | 0.09 | | -0.02 | | -0.07 | | 0.07 | | .27^**^ | | 0.08 | | -0.06 | | 0.03 | | 0.12 | | .16^*^ | | -0.12 | | -0.04 | | 0.03 | | .13^*^ | | .37^**^ | | 0.06 | | -0.08 | | 0.00 | | .15^*^ | | .16^*^ | | -0.09 | | -0.07 | | -0.11 | | .27^**^ | | (.87) | |  | |  | |  | |
| 28. Transfer of Competencies | 3.22 | 1.17 | 0.01 | | -0.08 | | .47^**^ | | .39^**^ | | .16^*^ | | 0.00 | | -0.01 | | .43^**^ | | .36^**^ | | .21^**^ | | 0.01 | | -0.04 | | .38^**^ | | .24^**^ | | .19^**^ | | -0.06 | | -0.03 | | .62^**^ | | .35^**^ | | .39^**^ | | -0.01 | | -0.02 | | .38^**^ | | .24^**^ | | .20^**^ | | 0.03 | | 0.00 | | (.86) | |  | |  | |
| 29. Transfer of positive Mood | 3.75 | 0.99 | -.14^*^ | | -0.12 | | .22^**^ | | .56^**^ | | .17^**^ | | -.17^**^ | | -.15^*^ | | .25^**^ | | .47^**^ | | .13^*^ | | -0.12 | | -0.02 | | .27^**^ | | .42^**^ | | .24^**^ | | -0.11 | | 0.03 | | .33^**^ | | .61^**^ | | .31^**^ | | -0.08 | | -0.03 | | .23^**^ | | .44^**^ | | .32^**^ | | -0.03 | | -0.01 | | .42^**^ | | (.89) | |  | |
| 30. Compen-sation | 3.49 | 1.23 | 0.03 | | -0.05 | | .21^**^ | | .20^**^ | | .39^**^ | | 0.00 | | -0.03 | | .20^**^ | | .29^**^ | | .25^**^ | | -0.07 | | 0.01 | | .29^**^ | | .20^**^ | | 0.11 | | -.32^**^ | | -0.06 | | .36^**^ | | .33^**^ | | .60^**^ | | -0.12 | | -0.07 | | .16^*^ | | .31^**^ | | 0.11 | | -.20^**^ | | -0.09 | | .39^**^ | | .21^**^ | | (.92) | |
| Note. * p < .05, ** p < .01; *N* = 277 | | | | | | | | | | | | | | | | | | | | | | | | | | | | | | | | | | | | | | | | | | | | | | | | | | | | | | | | | | | | | | |
